# Artificial Neural Networks in Classification of Steel Grades Based on Non-Destructive Tests

**DOI:** 10.3390/ma13112445

**Published:** 2020-05-27

**Authors:** Alexey Beskopylny, Alexandr Lyapin, Hubert Anysz, Besarion Meskhi, Andrey Veremeenko, Andrey Mozgovoy

**Affiliations:** 1 Department of Transport Systems, Faculty of Roads and Transport Systems, Don State Technical University, Gagarin, 1, 344000 Rostov-on-Don, Russia; 2 Department of Information Systems in Construction, Faculty of IT-systems and Technologies, Don State Technical University, Gagarin, 1, 344000 Rostov-on-Don, Russia; lyapin.rnd@yandex.ru; 3 Faculty of Civil Engineering, Warsaw University of Technology, Al. Armii Ludowej 16, 00-637 Warsaw, Poland; h.anysz@il.pw.edu.pl; 4 Department of Life Safety and Environmental Protection, Faculty of Life Safety and Environmental Engineering, Don State Technical University, Gagarin, 1, 344000 Rostov-on-Don, Russia; reception@donstu.ru (B.M.); mozgovoy.dstu@mail.ru (A.M.); 5 Department of Motor Roads, Faculty of Roads and Transport Systems, Don State Technical University, Gagarin, 1, 344000 Rostov-on-Don, Russia; veremeenko78@mail.ru

**Keywords:** non-destructive test, machine learning, clustering, steel, cone indentation, impact, artificial neural networks

## Abstract

Assessment of the mechanical properties of structural steels characterizing their strength and deformation parameters is an essential problem in the monitoring of structures that have been in operation for quite a long time. The properties of steel can change under the influence of loads, deformations, or temperatures. There is a problem of express determination of the steel grade used in structures—often met in the practice of civil engineering or machinery manufacturing. The article proposes the use of artificial neural networks for the classification and clustering of steel according to strength characteristics. The experimental studies of the mechanical characteristics of various steel grades were carried out, and a special device was developed for conducting tests by shock indentation of a conical indenter. A technique based on a neural network was built. The developed algorithm allows with average accuracy—over 95%—to attribute the results to the corresponding steel grade.

## 1. Introduction

The problem of assessing the mechanical characteristics of structural steels is an important task in engineering, construction, and other industries. Often in the construction site or manufacturing process, it is necessary to determine the grade of steel for subsequent processing. The most common laboratory tests for uniaxial tension, hardness testing, Charpy testing to determine the toughness are associated with the sample production and are practically not applicable to real structures. In the tasks of monitoring both the life and the safety of a structure, determining the strength class allows predicting reliability, especially if the structure has been working for a long time.

The resistance of materials to the action of the external factors is characterized not by the only one, but by a set of mechanical properties, which determines the strength, deformability, reliability, and quality of the structures. At the first stage, a set of the most important mechanical characteristics can be defined as the yield strength and ultimate strength of steel, elongation, and hardness. It is necessary to know these characteristics and be able to measure directly at the facility. Therefore, the task of developing methods and technical means for classifying structural steels according to strength groups, allowing quick and accurate measurement of the required indicators at any point in the real structure, is relevant and of considerable practical interest.

The most critical features of mechanical characteristics include the following three:

- (a) Random nature, which is their objective property and because of imperfections in the structure of the material. This circumstance requires the involvement of the apparatus of probability theory and mathematical statistics for the organization of control and certification of products;
- (b) The interdependence of mechanical properties, which requires the simultaneous determination of a set of strength characteristics on any part of the structure;
- (c) The dependence of mechanical properties on the loading history, which takes place in operation and transforms a random variable into a random function.

From the point of view of determining the mechanical characteristics, one of the most promising is the method of impact indentation. This method is non-destructive since for most designs the dimensions of the print do not affect the performance of the design

The relationship between the parameters of the indentation of the sphere with the strength characteristics of steel was established already in the works of Hertz et al., Brinell et al., Meyer et al., Tabor et al., and many others [1,2,3,4]. At the same time, the correlation dependences of the mechanical characteristics give too large errors. To more accurately determine the mechanical characteristics, other methods must be applied.

The models of interaction of indenters with various objects in most cases are based on an elastoplastic contact. On the one hand, this allows one to determine the elastic and plastic properties of the material; on the other hand, the nature of the interaction is in the non-linear deformation mode, which makes obtaining analytical dependences very difficult or impossible (Lee and Komvopoulos [5]). The exact solution can only be obtained in a linear setting. For example, Aizikovich et al. [6,7] considered the axisymmetric problem of pressing a rigid conical indenter into a half-space with a coating, the elastic moduli vary in depth. The statement of the problem in the framework of the linear theory of elasticity leads to an asymptotic solution of the double integral equation.

As a result, this caused the appearance of approximate or numerical methods for analyzing the contact interaction of bodies (Beskopylny et al. [8,9,10,11], Wang et al. [12], Trzepiecinski [13], Felipe-Sesé [14]). Yang and Komvopoulos [15] applied the finite element method to study the transition response of an elastoplastic layered medium pressed by a rigid cylinder and analyzed the reflection of stress waves from the layer/base interface depending on the indentation speed and indenter radius. Numerical methods, such as the finite element method or the method of boundary integral equations, allow us to visualize the results and analyze complex problems. Li et al. [16], developed the technique of boundary elements considering the adhesive and non-adhesive contact of arbitrary-shaped indenters with an elastic half-space. Numerical solutions are compared with the known asymptotic ones.

Patel et al. [17] considered another advantage of the finite element method, which consists in assessing the sensitivity of various assumptions and parameter values in the problem of indenting a spherical indenter in an inhomogeneous medium. Syngellakis et al. [18] simulated the interaction of a spherical indenter with an elastoplastic medium on the basis of real stress–strain curves. The influence of the contact interaction parameters on the characteristic indentation curves under active loading and unloading was studied, and the creep effect was analyzed.

Approximate analytical models and numerical methods coarsen the initial dependencies and relationships of mechanical characteristics, abstracting from, at first glance, less essential features. To more fully reflect the full depth of dependencies, artificial intelligence algorithms based on artificial neural networks, machine learning [19,20,21,22,23], and others began to be used. The relevance of the use of neural networks significantly increases when it becomes necessary to solve poorly formalized tasks, such as automation of the processes of classification, prediction, recognition, decision-making, an approximation of dependencies, etc.

Chun et al. [24], applied an approach in which the effective thickness of a shell element was estimated using a convolutional neural network (CNN), such as deep learning, performed on stretching structural elements. A neural network determines the characteristics of metal under uniaxial tension, and then the FEM model is built on the basis of a shell element that uses this effective thickness.

Machine learning algorithms (MLA) that were used to predict and classify the tensile strength of polymer films (Altarazi et al. [25]) and wire ropes (Kim et al. [26]) are considered. Multivariate experiments were designed with parameters taking into account the tensile test, and the tensile strength was recorded in good agreement with the predicted value. In both cases, the classification problems were combined with the known methods of FEM or statistical analysis.

A method for predicting the properties of ferrite-pearlite steels at cyclic stresses was proposed by Miyazawa et al. [27]. Numerical simulation by the finite element method was carried out using the analysis of the microstructure and properties of the material based on experimental results, and the cyclic tensile properties were calculated. Then, point-to-point correlations of synthetic microstructures were calculated to quantify fatigue strength. A study based on machine learning aimed at assessing the effect of paper shredding on fiber morphology was proposed by Almonti et al. [28]. In particular, an artificial neural network (ANN) was introduced, which was trained with experimental data to predict fiber lengths depending on the variables of the cleaning process.

A machine learning mechanism based on experimental data was used by Wu et al. [29] to develop models for predicting properties in order to reveal physical mechanisms and develop new materials with improved properties. To accurately predict and identify key features in determining the energies of hardening grain boundaries, three machine learning methods were used: A support vector algorithm with a linear core, an algorithm with a core radial basis function, and an artificial neural network.

Thus, it seems crucial to develop a method based on non-destructive testing methods that make it possible to evaluate a set of indentation characteristics that correlate with the material properties to classify steel grades at real structures. In this article, the technique is based on shock indentation tests of a conical indenter and the construction of an artificial neural network for classification of the results.

## 2. Materials and Methods

The destruction of structural elements is dynamic by its nature, so the reaction of the material to the impact application of the load is most impressive. The complexity of the processes that occur during dynamic testing of metals has led to many shock schemes of tests. An experimental study of the wave profiles of highly dispersed plastic waves was carried out by Bell et al. [30]. As a result, it was possible to compare the data obtained from the quasi-static analysis of uniaxial tension or compression with the region of high propagation velocities of plastic deformation waves of finite amplitude. The techniques of such experiments are quite complicated and do not allow their use in engineering practice.

In the practice of industrial production or construction, there are often cases of steel classification according to strength characteristics. At the same time, the number of such steel grades is limited since the construction firm deals with a limited number of classes of reinforcing bars, rolled products, or other units of high-grade metal. In this case, it is essential to have devices to express diagnostics and classification of steel. In our opinion, one of the promising areas is the method of determining the mechanical properties of shock indentation of indenters of various shapes. This approach allows the device to be compact and quickly identify the strength characteristics of steel anywhere in the structure.

The method of comparative determination of the Poldi-Hütte hardness, in which the hammer blow to the striker is transmitted to the standard tile and to the sample sandwiched between them by a ball or cylinder, is widely used (Matlin et al. [31]). However, the complexity of the automation of the measurement process makes such a device ineffective. Errors when using the Poldi-Hütte method can vary from 7 to 50% in various cases.

The hardness testing scheme is quite attractive when the indenter flies up to the measured surface with an initial velocity *V_0_*, and the ratio of approach and rebound speeds is measured. The devices of Leeb, Shor, Krautkramer, et al., are built on this principle. In such a scheme, various indenters were used: spherical, conical, pyramidal. Studies of German firms manufacturing instruments for measuring the hardness of materials have shown that such a test scheme for hardness is the most stable. At the same time, high stability and accuracy of measurements require a high degree of preparedness of the surface and a significant mass of the measured part. The test surface should be polished or finely ground and should not have scratches or other discontinuities that may affect the rebound characteristics out of the tested block. The final processing of the test surface should not exceed a maximum of 16 micro-inches (0.4 μm) [32], which significantly limits the use of the device in real operating conditions.

The large volume of control tests of materials in the industry poses the problem of moving from laboratory tests by the exemplary method to tests directly on the structure or in the workshops. Dynamic measurement methods meet such conditions.

Dynamic measurement methods, in our opinion, are preferred for the following reasons:

1. The dimensions of the device are significantly reduced, which makes it available in any part of the structure;
2. For most designs, dynamic loading is more dangerous than static;
3. The reaction of the material to the dynamic effect is more informative, which allows not only to evaluate the mechanical properties but also to develop effective technological operations (shot blasting, surface-plastic deformation, explosion, etc.);
4. φ change in the strain rate during testing can noticeably change the mechanical properties of the material, which is associated with a transition to another mechanical state (for example, in metals, from plastic-viscous to brittle; in polymers, from highly elastic to elastic). The appearance of a viscous-brittle fracture is extremely dangerous for metals since it leads to cracking and fracture. A viscous-brittle breach can only be predicted based on appropriate tests.

It is advisable to distinguish three main cases of the influence of the strain rate on the reaction of the material:

1. Low speeds (up to 10 m/s). It is generally accepted (Fridman [33]) that, in this case, wave processes and inertial forces do not significantly affect the reaction of the material and can be neglected. Most quasi-static impact models are applicable for this speed range. At the same time, it is incorrect to assume that the low strain rate does not affect the mechanical properties of the material. As experiments [34,35] show, the dynamic properties of the material (yield and strength limits, elongation and narrowing) significantly (up to two times) differ from static ones, which is associated with the processes of nucleation, development, and movement of dislocations, as well as with the diffusion rate chemical and physical processes, both inside the loaded body, and at the border with the environment.
2. High speeds (from 10 to 100 m/s). In this case, the inertial resistance of the material is substantial; the unevenness of the stress and strain states is great. The resistance of a material to pushing a solid striker into it increases in proportion to the square of the speed according to the law
  (1) $$\sigma =\sigma_{0}{\left(v/v_{0}\right)}^{n}+k_{0}\rho v^{2},$$
  where:$\sigma_{0}$—the resistance at low speed; $v_{0}$—static component; v—the speed of the striker; $k_{0}$ and *n*—empirical coefficients depending on the speed of the striker; ρ—the density of the half-space material.
3. Ultrahigh and hyper high speeds (from 100 m/s and above). At ultra-high speeds, the resistance increases even more, sometimes reaching an elastic modulus. It is generally accepted that at such rates, the hydrodynamic theory of flow is applicable to the material.

It is known that the strain rate during the test can significantly change the mechanical properties of the material. Therefore, in our case, it is desirable to use a speed range of up to 3 m/s. In this speed range, wave processes and inertial forces do not significantly affect the reaction of a steel sample and can be neglected.

**Figure 1:**
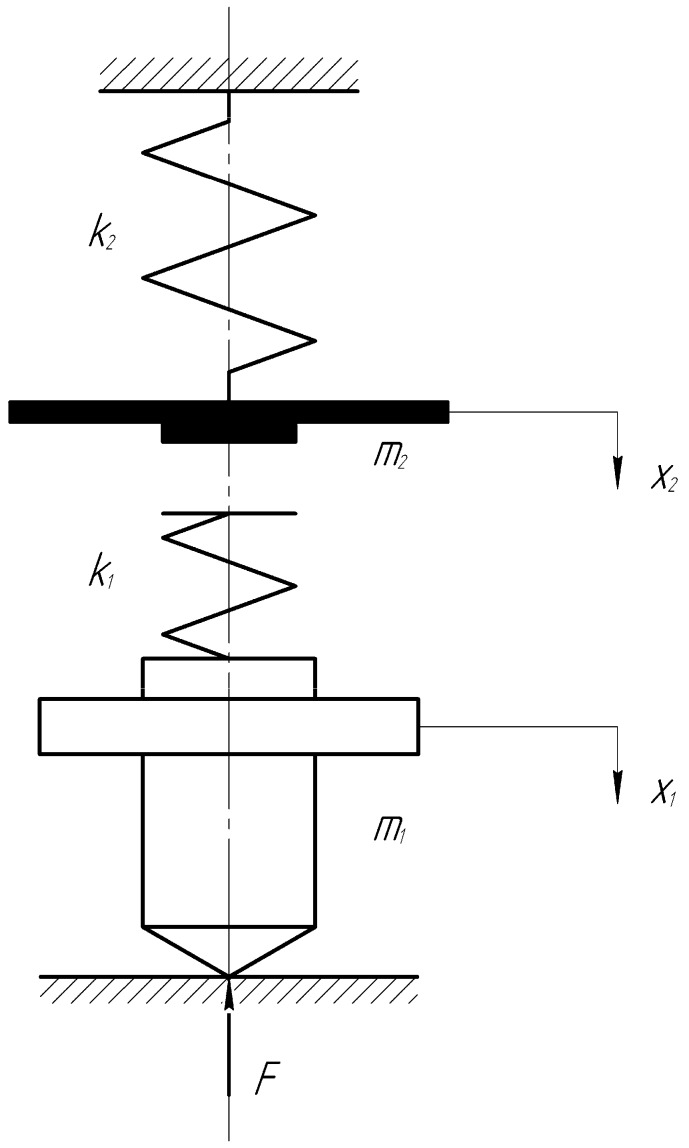
Scheme of the device for impact cone indentation.

In this work, we adopted a striking scheme (Figure 1), in which a tune of mass $m_{2}$ flies down with an initial velocity $v{|}_{t=0}=V_{0}$ to an initially motionless indenter of a mass $m_{1}$ of conical shape with a cone angle of 90°. In contrast to the well-known pattern of impact indentation of Leeb, in our case, the growth of stresses at the indenter tip starts from 0. It increases quite smoothly, which significantly affects the durability of the indenter during numerous tests.

**Figure 2:**
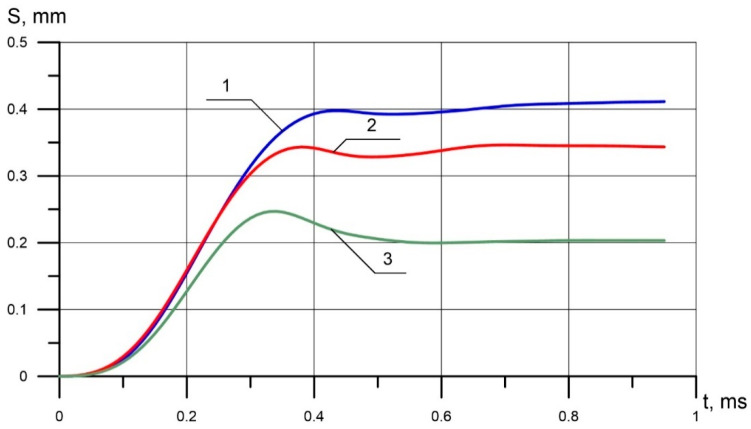
The relationship of the displacement of the conical indenter 90 on time during the impact: (**1**) 96 HB, (**2**) 182 HB, (**3**) 450 HB.

The stiffness of the main and auxiliary springs is $k_{2}$ and $k_{1}$, respectively. Strike approach speed $V_{0}$ = 2.5 m/s, impact energy *E* = 0.16 J (Figure 2).

Equations describe the motion of the elements of the system (Figure 1)

(2) $$\begin{array}{c} m_{1}{\ddot{x}}_{1}=k_{1}\left(x_{2}-x_{1}\right)-F\left(t\right)\\ m_{2}{\ddot{x}}_{2}=k_{2}\left(x_{st}-x_{2}\right)-k_{1}\left(x_{2}-x_{1}\right) \end{array}$$

at $x_{1}=0,\;x_{2}=0,\;{\dot{x}}_{1}=0,\;{\dot{x}}_{2}=V_{0}$, $x_{st}=const$.

The force of resistance of the material to the indentation of the indenter *F*(t) depends on the mechanical characteristics of the material and is non-linear. During the impact, the displacement S(t), the velocity V(t), and the acceleration W(t) of the indenter are recorded. The analytical solution of the inverse problem of determining the mechanical characteristics of steel from the experimentally measured curves S(t), V(t), and W(t) is complicated. Therefore, in the present work, the first stage was completed—the classification of steels based on ANN algorithms. Figure 2 shows the experimentally obtained dependence of the displacement of the conical indenter 90° on time during the impact.

**Figure 3:**
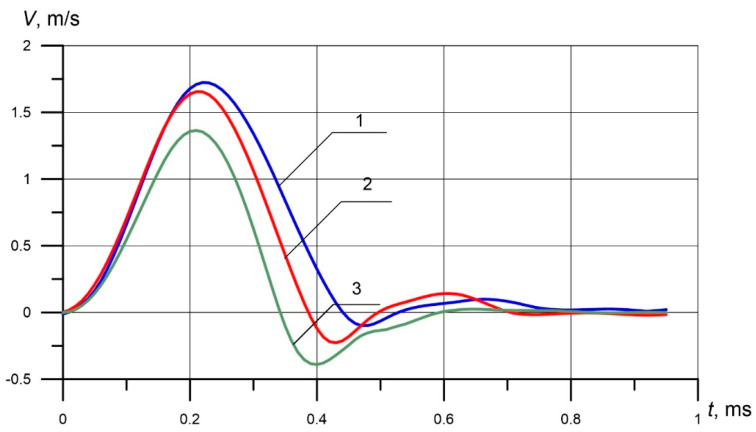
The relationship of the velocity of the conical indenter 90 on time during the impact: (**1**) 96 HB, (**2**) 182 HB, (**3**) 450 HB.

It is seen that at the beginning of the contact, the indenter is on the surface of the part, and its speed is zero (Figure 3). The striker *m_2_* (Figure 1) flies up to the indenter, accelerates it upon impact, and the indenter further moves deeper into the material in two stages. The active stage, when the indenter accelerates, and its speed reaches its maximum value (Figure 3), then under the influence of the resistance forces of the material, the velocity drops to 0. In the passive stage, the indenter rebounds. At this stage, the indenter speed becomes negative (Figure 3) and gradually oscillates at a level of 0.

**Figure 4:**
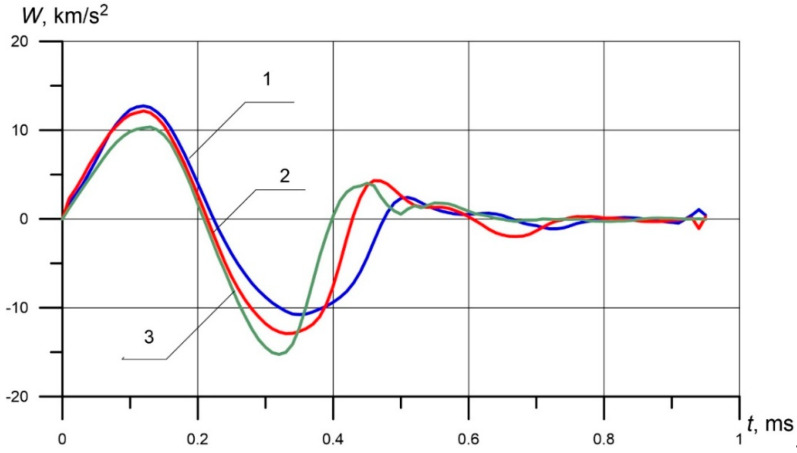
The relationship of the acceleration of the conical indenter 90 on time during the impact: (**1**) 96 HB, (**2**) 182 HB, (**3**) 450 HB.

Figure 4 shows the acceleration of the indenter during impact. It is seen that in the active phase when V(t)≥0 the indenter acceleration has two characteristic extremums: the maximum value of acceleration in the positive region when the indenter accelerates (Figure 4), and the minimum amount in the process of deceleration of the indenter.

Both phases of the indenter movement, active and passive, under constant conditions of impact, are determined by the properties of the material.

Structural carbon steels widely used in engineering and construction 08 kp, 18KhGT, 20, 35, 40Kh, 45, 50KhFA, 65G were selected as test materials in the hardness range 96 HB - 450 HB. The chemical composition of the steels is given in Table 1.

**Figure 5:**
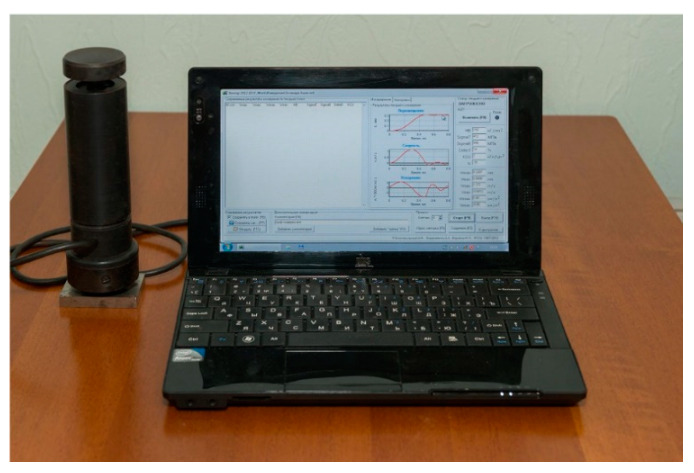
The device for impact cone indentation.

The implementation of the proposed experimental research algorithm in the form of a device for impact testing of materials is shown in Figure 5. In the device, the spring is charged manually by lifting the handle up. Subsequent pressing on the handle actuates the spring, and an impact is made to the motionless indenter.

For the 9 classes of steel (Table 1), 67 samples were manufactured, and 67 tests were carried out. Samples were not heat treated and tested in the delivery state. The surface of the samples was ground to a depth of 0.1 mm to remove oxides and corrosion. During the test, the conical indenter hits on the steel sample, and the indenter velocity was measured. The acceleration was obtained by differentiating the velocity function, and the indenter movement was measured by integration of the velocity curve. The approximate time of one test continued for 0.95 ms, with quantization frequency 100 kHz. For each sample, several strokes were applied; the average value was taken to the database. These were the basis for the database created presented in Appendix A.

There are 95 accelerations values for each test, and they are measured every 0.01 ms. Each steel element (one for each class) was tested from 5 to 12 times with different positions of the measuring device (horizontal, vertical, location on a steel element).

## 3. Results

**Figure 6:**
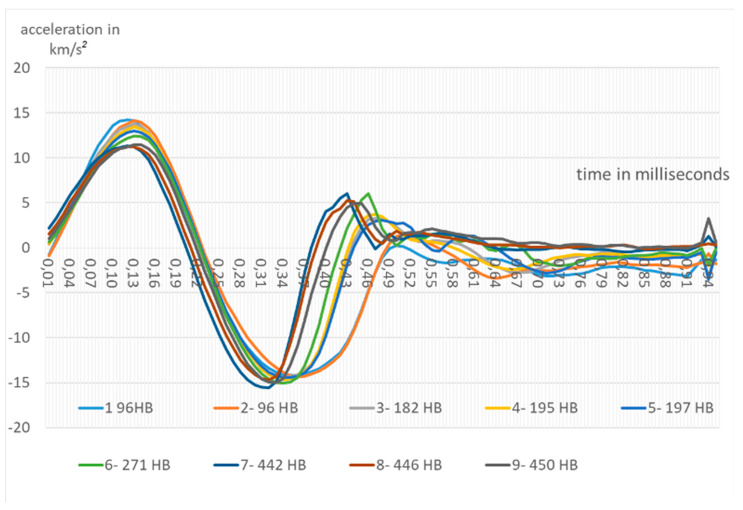
Experimental dependences indenter acceleration during of the shock indentation.

Acceleration W(t) is given in km/s^2^, and the sign (-) means a slowdown of indenter into the examined steel element. The measured accelerations for selected 9 experiments (out of 67) are presented in Figure 6.

The analysis of the intender movement’s parameters is confirmed by the theoretical analysis [10]. However, the attempt to predicting the class of the examined steel element was not a subject of [10].

**Figure 7:**
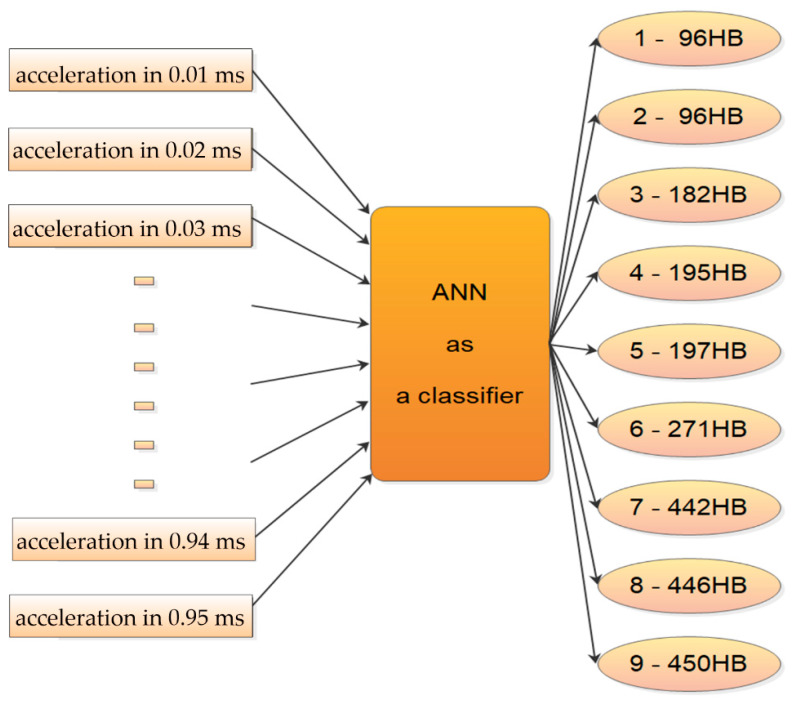
The scheme of classifying into nine classes of steel based on the intender acceleration.

Based on many examples of successful application of machine learning in civil engineering problems’ solving [36,37,38,39], artificial neural networks are chosen for automatically classifying the type of the steel based on the acceleration of cone intender. ANNs’ classifying features are widely proved e.g., in [40,41,42,43]. Moreover, examining features of construction materials are the subject of many kinds of research e.g., [21,44,45,46,47]. A low number of recorded cases for the machine learning application is questionable. However, the attempt to automatic classification is also this time successful, and it is described hereinafter. The examples of the ANN successful application with a relatively low number of records in the database—achieved through fine tuning of ANN parameters—can be found e.g., [48]. The ANN’s ability to classify is based on its properties to find—with metaheuristic algorithms—the set of so-called weights. These weights are the part of the transformation of the input signal into the output in all layers of ANN (multilayer perceptron type) except in the input layer. The aim of finding the weights is to minimize the error at the output for all samples (consisting of several input data types). The scheme of ANN acting as a classifier is presented in Figure 7.

In the classification problem, results are measured through, the so-called, confusion matrix. The number of correctly classified cases and incorrectly classified (as well as their share within cases belonging to a specific class) are presented there. To achieve a high level of correct classifications, the database should be carefully prepared. The values of acceleration are standardized with the use of min-max method. The following formula based on [22,49] is applied:

(3) $$w_{i1}=\frac{w_{i0}-\underset{i}{\min}\left(w_{i0}\right)}{\underset{i}{\max}\left(w_{i0}\right)-\underset{i}{\min}\left(w_{i0}\right)}$$

where: $w_{i0}$: the acceleration of the intender in a given test before standardization; $w_{i1}$: the standardized acceleration of the intender in a given test; i: test number, integer value from 1 to 67.

This type of standardization allows for avoiding negative values in the dataset. The full database **W** consist of 67 rows (tests), and 95 columns (95 accelerations measured in one test every 0.01 ms). The symbol $w_{5,\;10}$ means acceleration in the 5th test, measured in 0.10 ms. As there is a relatively low number of tests (for machine learning purposes), the random choice of the cases to the three subsets (training, testing, and validating) is not applied. The risk of omitting any sample from a certain class in any of the subsets is avoided. It was the authors’ decision which sample served for training ANN, which samples were used for testing purposes (protection against overfitting), and which samples belong to the validating subset—necessary to create the confusion matrix. Samples from each class of steel are present in every subset of input data. The software used for ANN calculation Statistica 13.1 (by Dell, Round Rock, TX, USA) allows for such manual dividing data. Suggested ratios of samples in each subset is 60:20:20 or 70:15:15 (e.g., in [50,51]) is almost kept by assigning 45 cases to the training dataset, 11 to the testing, and 11 to the validating dataset. This kind of supervised division of data onto three subsets was successfully applied in [43], where one class had a really low number of samples. To provide reliable results of an automatic classification, the results from ANN are given as average from six-folds cross-validation. Even then, when the cross-validation is applied, the choice of the samples to the three subsets is not random. The assignment of all samples from class 3 182 HB to the subsets is presented in Table 2 as an example. The distribution process is similar for the other eight classes.

During the calculation process, it was clear from the very beginning that the number of inputs (95 accelerations of the intender measured for each case) is too large when compared to the number of records in the database (67) [52,53]. It is assumed that the bigger the range, the easier ability to recognize which group of tests a given value of acceleration belongs to. The ranges r of measured accelerations w are calculated at each time instant:

(4) $$r_{j}=\left|\underset{i}{\max}\left(w_{ij}\right)-\underset{i}{\min}(w_{ij})\right|$$

where j is the number of the measurement in one test (1≤j≤95).

As it is not sure which phase of movement of the intender can be a best for recognizing the class of steel, it is decided to divide the time of the intender movement into ten segments. The first one starts at 0.01 ms and ends at 0.10 ms, the second one is 0.11–0.20 ms interval, etc., and finally, 0.91–0.95 ms interval creates the last segment. To limit the number of weights in ANN (achieved by limiting the number of inputs), from each segment, one time instant was selected based on the maximum range $r_{j}$ (found for this segment). The input to ANN is limited then to ten features F (one per segment), and it can be expressed as:

(5) $$Input\;=\;\left[\begin{array}{c} F^{\left(1\right)}:\;\;\underset{j}{\max}(r_{j}^{\left(1\right)})\;\;\;\;\;\;\;for\;\;1\;\;\leq j\leq 10\;\\ F^{\left(2\right)}:\;\;\underset{j}{\max}(r_{j}^{\left(2\right)})\;\;\;\;\;\;\;for\;\;11\leq j\leq 20\;\\ F^{\left(3\right)}:\;\;\underset{j}{\max}(r_{j}^{\left(3\right)})\;\;\;\;\;\;\;for\;\;21\leq j\leq 30\;\\ \vdots \\ F^{\left(9\right)}:\;\;\underset{j}{\max}(r_{j}^{\left(9\right)})\;\;\;\;\;\;\;for\;\;81\leq j\leq 90\;\\ F^{\left(10\right)}:\;\;\underset{j}{\max}\left(r_{j}^{\left(10\right)}\right)\;\;\;for\;\;91\leq j\leq 95\; \end{array}\right]\;\;$$

It is calculated that the highest ranges of accelerations are at: 0.07, 0.20, 0.23, 0.40, 0.42, 0.60, 0.61, 0.71, 0.90, 0.94 ms. The accelerations from these moments created the input database. The software allows for the automatic searches for the number of neurons in the hidden layer of ANN, the type of activation function in the hidden layer, and in the output layer, the type of metaheuristic algorithm for searching the optimum set of weights. The following procedure is applied for each fold:

- The automatic search for ten of the best classifying networks (out of 1000 found);
- The manual choice, at least eight ANNs (out of ten proposed) providing a high level of correct classification in every subset (training, testing and validating);
- Making predictions for all 67 cases based on the ensemble of the chosen networks (retaining information which case belongs to each subset).

The classifications by the ensembles of ANN from each fold for the validation subset are presented in Table 3. False results are highlighted in red there.

The average level of the correctly classified cases is 80.3% for the validation dataset. For all subsets the result is and equal to better 89.3% but in fact its meaningless, as in the practice the user would rely on prediction made for unknown class of still. In the worst case 5/11 of automatic classifications can be false (see Fold 6 in Table 3). To increase the accuracy, the number of inputs was limited (in different configurations) and the described above procedure was repeated. It has not produced better correctness of classifications.

**Figure 8:**
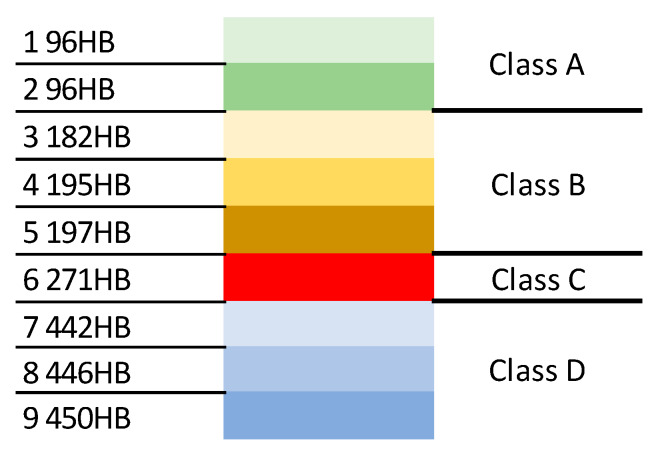
The original and the new classes with the colures assigned.

Expecting that the proposed method should bring much better results the set of data is analysed again. Based on misclassified steel classes (presented in Table 3) the original nine classes of steel are grouped into four new classes (A, B, C, and D) to avoid misclassifications when the new classes are considered. Original and new classes are presented in Figure 8.

**Figure 9:**
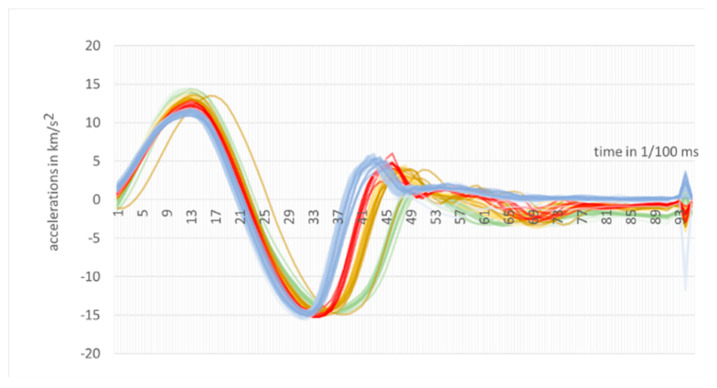
All accelerations of 67 tests, measured in 95 moments (in each test).

The colours assigned to the classes are then applied for creating the curves of accelerations simultaneously for all tests as presented in Figure 9.

**Figure 10:**
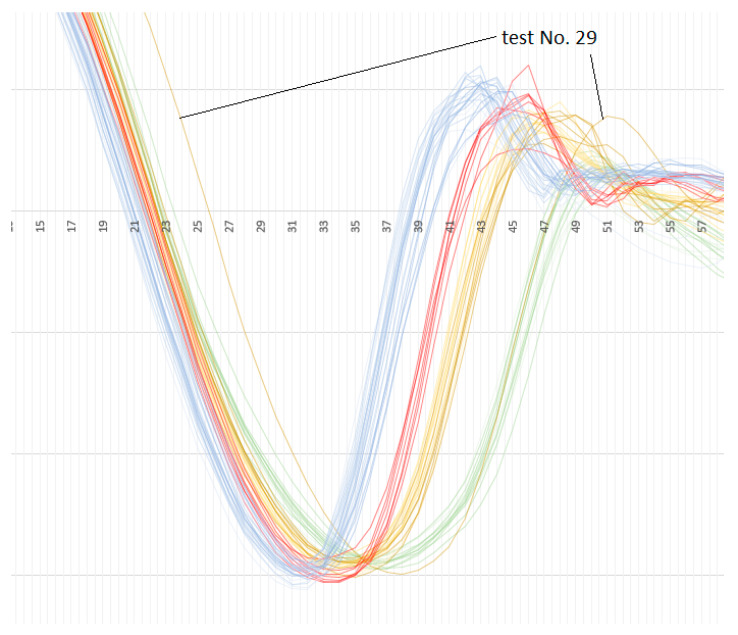
The curve of the test No. 29—out of phase.

It can be observed in Figure 9 that accelerations of one test from class 5 197 HB are out of phase. The closer look proves that this dark yellow (or light brown) line goes among green lines within the interval 0.45 – 0.50 ms (see Figure 10). It is verified that these are the results of the test number 29 (see Appendix A).

**Figure 11:**
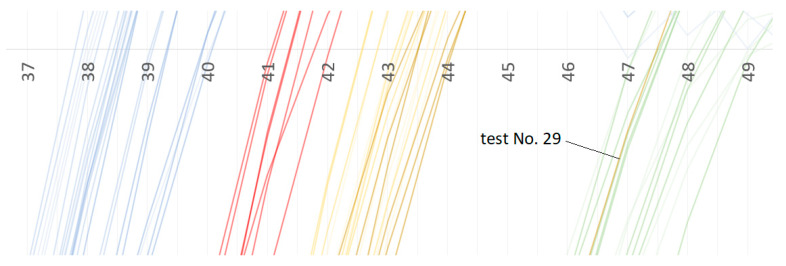
The curve of the test No. 29—out of phase, zoomed in.

The next important observation is that the new classes can be easily distinguished within the interval of 0.33–0.50 ms. However, as it is visible in Figure 11, the sequence of classes (presented in Figure 8) is not kept.

These findings are the base of the decision to exclude this record (accelerations measured in test No. 29) from the database. To repeat the calculations, the input data have to be prepared with the same procedure. The choice of ten features (10 inputs) has to be repeated as the ranges of acceleration in certain moments were disturbed by the acceleration of the sample No. 29. It has occurred that only two input variables are changed (the first one—to 0.02 ms, and the third one to 0.22 ms). The procedure of finding the best ANN set for classifying steel grades into nine classes is applied again. It has produced slightly better averaged (from cross-validation) results i.e., 83.3% of correct classification for the validating dataset (and 86.6% for all 66 tests, as test No. 29 is excluded from analysis). The correctness of 83.3% still means 11 tests falsely classified for all 66 from six folds (for the validating dataset).

While analyzing the curves presented in Figure 9, Figure 10 and Figure 11, and based on the full accelerations presented in Appendix A a simple, reliable classifier can be created, if the sample No. 29 is excluded. It divides all 66 tests in to four class A, B, C, and D with correctness of 100%.

(6) $$\mathrm{class}=\{\begin{array}{c} {A\;\mathrm{if}\;w}_{i}\;\mathrm{changes}\;\mathrm{the}\;\mathrm{sign}\;\mathrm{to}+\mathrm{within}\;\mathrm{interval}\;0.370-0.405\;\mathrm{ms}\\ {B\;\mathrm{if}\;w}_{i}\;\mathrm{changes}\;\mathrm{the}\;\mathrm{sign}\;\mathrm{to}+\mathrm{within}\;\mathrm{interval}\;0.406-0.423\;\mathrm{ms}\\ {C\;\mathrm{if}\;w}_{i}\;\mathrm{changes}\;\mathrm{the}\;\mathrm{sign}\;\mathrm{to}+\mathrm{within}\;\mathrm{interval}\;0.424-0.450\;\mathrm{ms}\\ {D\;\mathrm{if}\;w}_{i}\;\mathrm{changes}\;\mathrm{the}\;\mathrm{sign}\;\mathrm{to}+\mathrm{within}\;\mathrm{interval}\;0.460-0.495\;\mathrm{ms} \end{array}$$

Based on that, it is decided to check if ANN with inputs from the range 0.37 to 0.50 ms is able to correctly classify into nine classes. The following set of moments are chosen: from 0.38 to 0.44 ms, and 0.47, 0.48, 0.49 ms. It takes ten inputs into ANN. The results achieved this time are worse than in the previous two attempts to creating and running the automatic classifier. The correct classifications are in 69.7% of the test from the validating datasets (as an average from six folds of the cross-validation process). For all the tests from the three subsets, the average correctness is 69.4%.

**Figure 12:**
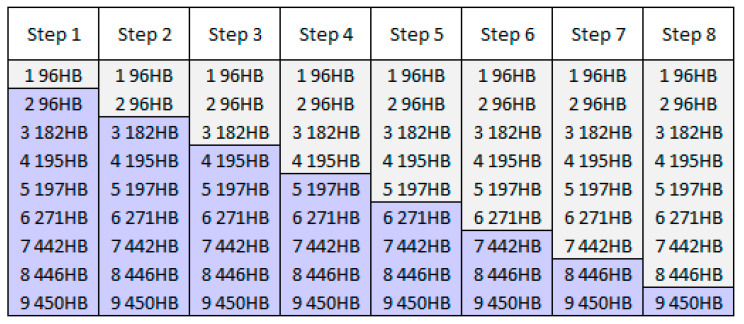
The step classification scheme into two subsets, grey and violet at every step.

Based on the improper balance of the number of weights values to be found (during searching for the best classifying ANN) through metaheuristic algorithms and the number of tests in the input database, it was decided to search a single border between every two neighboring classes. In can be calculated through classifying into two classes only, but eight times consecutively i.e., in eight steps (see Figure 12).

This approach makes the number of weights (between the hidden layer and the output layer of ANN) lower when compared to the nine classes applied at the output. To find the best classifying ANNs, the same division onto the three subsets is used, as well as before the cross-validation is applied (six folds). It should be noticed that the number of tests in grey and violet classes can differ much (extremally in Step 1 and Step 8). It is expected that test not belonging to the originally neighboring classes (like e.g., a sample from class 2 96 HB and a sample from class 7 442 HB, in Step 4) will be easily and correctly distinguished by ANN as a sample belonging to grey or violet class. The crucial are classifications of the tests belonging to originally neighboring classes (like e.g., to classes 5 197 HB and 6 271 HB in Step 5). But this implies the specific form of presentation of the results achieved. The results traditionally presented are summarized in Table 4.

The results seem impressive, and they really are, but only for Step 1, Step 2, and Step 6. But the results in the other steps are not so optimistic as presented. Considering classification in Step 8, in can be read in Table 5 that despite the fact that classifications are to grey and violet classes for the total number tests in class 9 450 HB i.e., 42, more than 30% (13 tests) are incorrectly classified. Does this really make the usefulness of the proposed method low? Let us analyze the numbers of correctly and incorrectly classified tests from all three subsets (summarized from all six folds) considering the original classes. In Table 5, the incorrect classifications are presented in red. Horizontal lines there represents the division of classification in a particular step (the grey class is above the line, the violet one, below the line).

Except for the classification in Step 7, if falsely classified tests appear, they belong to the original class the closest to the division between two output classes in a given step. Only in Step 7 and Step 8 false classifications appear at “grey” and “violet” classes. The way of applying the automatic classification tool to the new test, together with assessing of the model is presented in the following discussion section.

## 4. Discussion

The created tool consists of eight separate automatic classifiers. Assessing the class of the steel for the new case (based on the set of accelerations achieved from the device described), the ten (specified before) accelerations should be chosen and standardized with the same method as the one applied in the article. Then, eight classifications could be done, starting from the tool called Step 1, and ending at Step 8. Based on the assumed results for this sample, the following reasoning can be conducted:

- If the result from Step 1 classified the sample to grey class, the class of steel is 1 96 HB with 100% confidence (it stops the reasoning), if not, take Step 2;
- If the result from Step 2 classified the sample to grey class, the class of steel is 2 96 HB with 100% confidence (it stops the reasoning), if not, take Step 3;
- If the result from Step 3 classified the sample to grey class, the class of steel is 3 182 HB with 100% confidence (it stops the reasoning). Five out of 42 classifications in Step 3 are incorrect i.e., falsely classified to 4 195 HB (but at this moment, it is assumed that Step 3 classified to the grey class; it stops the procedure). If Step 3 classified the sample to the violet class, take Step 4;
- If the result from Step 4 classified the sample to grey class, the class of steel is 4 195 HB with $\frac{42}{42+6}=87.5\%$ confidence (it stops the procedure). In six cases out of 48 it can belong to 5 197 HB. If the sample is classified to the violet class, go to Step 5;
- If Step 5 classified the sample to the grey class, the sample is 5 197 HB in 100% confidence (there is one case falsely classified to the violet class, but for this moment it is assumed that Step 5 classified the sample to the grey class); if Step 5 classified the sample to the violet class, go to Step 6;
- If Step 6 classified the sample to the grey class, the sample is 6 271 HB in 100% confidence, if not, go to Step 7;
- If Step 7 classified the sample to the grey class, the sample is 7 442 HB with $\frac{29}{29+1}=96.7\%$ confidence (it stops the procedure). This one case from class 9 450 HB can be classified as belonging to the grey class. If the sample is classified to the violet class, go to Step 8;
- If Step 8 classified the sample to the grey class, the sample is 8 446 HB with $\frac{69}{69+13}=84.1\%$ confidence, if not (the class pointed by Step 8 is violet) the sample belongs to 9 450 HB with confidence equals to $\frac{29}{29+3}=90.6\%$ (this ends the procedure).

The step procedure of classifications onto two classes in one step allows for the kind of reasoning, where 5 out of 9 classes are assigned to the new sample (for which the class of steel is unknown) the correct class with 100% confidence. The level of confidence for each class is presented in Table 6.

The 8 steps procedure significantly increased the possibility of assessing the class of steel based on accelerations. According to the manual assessing (based on rough data and their visual expressions) only four, wide classes can be distinguished with the confidence of 100%. One of them is class C, i.e., the class 6 271 HB. The other, original classes could not be distinguished in this method—only groups of them (see Figure 8). Applying ANN for automatic classifying into nine classes produced as average 83.3% correct classifications for validating dataset, and 86.6% for all tests. Reading the numbers—averaged results of classifying in each step (see Table 4)—it can be assessed that they are the best. However, when analyzed in detail—the correctness of crucial tests (lying close to the border) is much lower compared to the overall correctness in a certain step. This problem is overcome by the 8-step reasoning (described above). It allows, based on 66 tests for determining (i.e., with confidence 100%) the membership of originally unknown test to five classes. The confidence of the membership to the other four classes is between 84.1% and 96.7% (depending on class), as presented in Table 6.

The proposed method is an intensive extraction of knowledge from the database, which consists of a low number of records. The minimum number of tests in one class is five tests; the maximum is 12. This is to emphasize that reasoning based on that is very sensitive for any new test added to the database. They are highly desirable to improve the statistical meaning of the proposed method and the confidence of automatic classifications. Despite the higher number of tests for training the ANN, another issue, which could increase the accuracy of the method, is the information about the direction of the device during testing a steel element. The results may vary when the gravity is subtracted from or added to the measured acceleration. Also, the friction between the moving intender and the pipe limiting its movement is different in the vertical and the horizontal position of the device during the measurements. These issues will be a subject of future researches.

## 5. Conclusions

Based on the close correlation between calculated and observed parameters of movement, of the cone intender of the invented tool for steel elements examination, the article is an attempt to automatically determine the steel type, based on the recorded movement of the intender. With the use of ANN (MLP type) the sequence of the observed acceleration of the indenter is analyzed, and based on that, the set of observations is classified. There are too many observations of accelerations when compared to the number of records in the database. Based on the highest range of accelerations, ten inputs are chosen. Originally nine classes of steel are applied as an output. The level of correct classifications for the validating dataset achieved is 83.3%, and 86.6% for all tests. The not satisfactory result forced the application of eight-step ANN classifying to two classes only in each step. This method significantly improved the confidence of reasoning based on the invented tool when a new test is assessed. The automatic, eight-step classifications provide the highest confidence of the membership of a new sample to one of five classes. If the application of the invented tool points the membership to one of the other four classes, these should be assessed with the confidence from 84.1% to 96.7%. The average confidence achieved for the model is 95.4%. The use of ANN for classification significantly increased the accuracy of assessment of steel grades based on accelerations measured with the measuring device with cone intender. The set of information gathered in one test is much higher than the possibility of analyzing it with machine learning tools. This is according to a relatively low number of records in the database (66 tests). Nevertheless, the presented analysis proved that the proposed method is able to recognize the steel grade with the accuracy sufficient for practical, engineer’s expertise. Increasing the number of tests, repeating them with the same conditions, and recording the spatial position of the measuring device may increase the accuracy of the method and simplify calculations.

## Figures and Tables

**Table 1:** The chemical composition of steels adopted for experimental research.

Steel	C/%	Si/%	Mn/%	Ni/%	Cr/%	Cu/%	Ti/%	V/%	GOST
08 kp	0.05–0.11	0.03	0.25–0.5	0.25	0.1	0.25			1050
09G2C	<0.12	0.5–0.8	1.3–1.7	0.3	0.3	0.3			19282
18KhGT	0.17–0.23	0.17–0.37	0.8–1.1	0.3	1.0–1.3	0.3	0.03–0.09		4543
20	0.17–0.24	0.17–0.37	0.35–0.65	0.25	0.25	0.25			1050
35	0.32–.04	0.17–0.37	0.5–0.8	0.25	0.25	0.25			1050
40Kh	0.36–0.44	0.17–0.37	0.5–0.8	0.3	0.8–1.1	0.3			4543
45	0.42–0.5	0.17–0.37	0.5–0.8	0.25	0.25	0.25			1050
50KhFA	0.46–0.54	0.17–0.37	0.5–0.8	0.25	0.8–1.1	0.2		0.1–0.2	14959
65G	0.62–0.7	0.17–0.37	0.9–1.2	0.25	0.25	0.2			14959

**Table 2:** Distribution of samples from class 3 182 HB for T—training subset, U—testing subset, and V—validating subset, for the cross-validation purposes.

Sample Number	Fold 1	Fold 2	Fold 3	Fold 4	Fold 5	Fold 6
15	U	T	T	U	T	T
16	V	T	T	T	T	T
17	T	U	T	V	U	T
18	T	V	T	T	T	T
19	T	T	U	T	V	U
20	T	T	V	T	T	T
21	T	T	T	T	T	V

**Table 3:** Result of classifications by an ensembles of artificial neural networks (ANNs) for each fold.

Original Class	Predictions					
	Fold 1	Fold 2	Fold 3	Fold 4	Fold 5	Fold 6
1 96 HB	1 96 HB	1 96 HB	2 96 HB	1 96 HB	1 96 HB	2 96 HB
2 96 HB	2 96 HB	2 96 HB	2 96 HB	2 96 HB	2 96 HB	2 96 HB
2 96 HB	2 96 HB	2 96 HB	2 96 HB	2 96 HB	2 96 HB	2 96 HB
3 182 HB	3 182 HB	3 182 HB	3 182 HB	3 182 HB	3 182 HB	5 197 HB
4 195 HB	3 182 HB	4 195 HB	5 197 HB	4 195 HB	4 195 HB	5 197 HB
5 197 HB	5 197 HB	4 195 HB	4 195 HB	5 197 HB	5 197 HB	4 195 HB
6 271 HB	6 271 HB	6 271 HB	6 271 HB	6 271 HB	6 271 HB	6 271 HB
7 442 HB	9 450 HB	7 442 HB	7 442 HB	9 450 HB	8 446 HB	8 446 HB
8 446 HB	8 446 HB	8 446 HB	8 446 HB	8 446 HB	8 446 HB	8 446 HB
8 446 HB	8 446 HB	8 446 HB	8 446 HB	8 446 HB	8 446 HB	8 446 HB
9 450 HB	9 450 HB	9 450 HB	9 450 HB	9 450 HB	9 450 HB	9 450 HB

**Table 4:** Averaged results of classifications of ANNs in each step.

Subset	Average Correctness Level in the Steps (in/%)							
	Step 1	Step 2	Step 3	Step 4	Step 5	Step 6	Step 7	Step 8
validating	100.0	100.0	98.0	95.5	99.7	100.0	96.2	95.5
all subsets	100.0	100.0	100.0	100.0	100.0	100.0	97.0	95.5

**Table 5:** Result of step classifications for all subsets summarized from six folds.

Original Class	Number Correct and Incorrect Classifications							
	Step 1	Step 2	Step 3	Step 4	Step 5	Step 6	Step 7	Step 8
1 96 HB	30	30	30	30	30	30	30	30
2 96 HB	54	54	54	54	54	54	54	54
3 182 HB	42	42	37; **5**	42	42	42	42	42
4 195 HB	42	42	42	42	42	42	42	42
5 197 HB	36	36	36	30; **6**	35; **1**	36	36	36
6 271 HB	42	42	42	42	42	42	42	42
7 442 HB	42	42	42	42	42	42	29; **13**	42
8 446 HB	72	72	72	72	72	72	72	69; **3**
9 450 HB	42	42	42	42	42	42	41; **1**	29; **13**

**Table 6:** Result of classifications by an ensemble of ANNs for each fold.

End of Assessment at	Class Assessed	Confidence of Assessment	Possible Faults
Step 1	1 96 HB	100.0%	None
Step 2	2 96 HB	100.0%	None
Step 3	3 182 HB	100.0%	None
Step 4	4 195 HB	87.5%	5 197 HB can be classified as 4 195 HB
Step 5	5 197 HB	100.0%	None
Step 6	6 271 HB	100.0%	None
Step 7	7 442 HB	96.7%	9 450 HB can be classified as 7 442 HB
Step 8	8 445 HB	84.1%	9 450 HB can be classified as 8 445 HB
	9 450 HB	90.6%	8 445 HB can be classified as 9 450 HB

## Appendix A

**Table A1:** Accelerations measured for tests from 1 to 14.

Time in ms	1 96 HB					2 96 HB								
	Test 1	Test 2	Test 3	Test 4	Test 5	Test 6	Test 7	Test 8	Test 9	Test 10	Test 11	Test 12	Test 13	Test 14
0.01	−0.3508	−1.2392	−0.7800	−0.3720	−0.7948	0.3948	−1.3889	−0.7233	−0.2569	−0.8946	−0.1574	−0.6031	−0.1621	−0.7674
0.02	0.8505	0.2169	0.9772	1.3952	0.5525	1.8073	−0.3889	0.7866	1.0193	0.6470	0.9928	0.8598	1.1990	0.6386
0.03	2.3081	1.8648	2.7355	3.2623	2.1400	3.4737	0.8846	2.3011	2.4683	2.1594	2.3879	2.3138	2.7697	2.0471
0.04	4.0649	3.7459	4.5665	5.2294	3.9830	5.3565	2.4212	3.8948	4.1433	3.7607	4.0571	3.8592	4.5581	3.5759
0.05	5.8770	5.7252	6.4610	7.0958	5.7402	6.8921	4.0692	5.6010	5.6715	5.5750	5.5447	5.5855	6.1406	5.2862
0.06	7.8200	7.6150	8.2141	8.8518	7.4882	8.4192	5.7667	7.2728	7.2566	7.1956	7.0766	7.2469	7.7005	6.8505
0.07	9.7782	9.4424	9.8919	10.5500	9.2761	10.0778	7.4713	8.8498	8.9049	8.7501	8.7189	8.8507	9.3372	8.4491
0.08	11.2408	10.9870	11.4036	11.9153	10.6403	11.2272	9.0468	10.2758	10.1794	10.2998	9.9402	10.3225	10.6131	9.9714
0.09	12.4967	12.2547	12.5650	13.0021	11.8679	12.3611	10.4838	11.4529	11.3794	11.4537	11.1498	11.4954	11.8388	11.2015
0.10	13.5303	13.3822	13.5697	13.9052	12.9176	13.2439	11.7424	12.4812	12.3719	12.5519	12.1794	12.5813	12.8921	12.4534
0.11	14.0830	14.0312	14.1076	14.3014	13.4789	13.6249	12.6816	13.1811	12.9867	13.3765	12.7508	13.3403	13.4690	13.3283
0.12	14.4026	14.3853	14.1923	14.3610	13.8520	13.9797	13.3673	13.6478	13.4576	13.7732	13.2762	13.8063	13.9224	13.9195
0.13	14.3485	14.4396	14.1381	14.2186	13.8872	13.8809	13.8853	13.9450	13.6332	14.1298	13.5053	14.1770	14.1208	14.3962
0.14	13.8763	13.8507	13.5035	13.4419	13.4531	13.2876	13.9832	13.6443	13.3486	13.9755	13.2832	13.9750	13.7603	14.2588
0.15	13.1391	12.9529	12.5113	12.3016	12.7237	12.3974	13.7171	13.0927	12.7166	13.3392	12.8608	13.4170	13.0586	13.8311
0.16	11.9428	11.7584	11.2767	10.8988	11.5043	11.0035	13.0962	12.1645	11.6494	12.4444	11.9743	12.5036	11.9368	13.0117
0.17	10.3662	10.1259	9.5892	9.0303	9.9132	9.4642	11.9338	10.7371	10.2645	10.9936	10.6956	11.0684	10.4768	11.5979
0.18	8.6348	8.4701	7.8503	7.2157	8.2931	7.8269	10.5571	9.3761	8.8065	9.3850	9.3225	9.6272	8.9571	10.1864
0.19	6.6833	6.5507	5.9265	5.2652	6.4434	5.8484	8.9920	7.6853	7.1061	7.6878	7.6431	7.9364	7.1844	8.4579
0.22	4.6213	4.3220	3.7560	3.0096	4.4085	3.8055	7.1724	5.6273	5.2234	5.6248	5.7770	5.8720	5.1271	6.3478
0.21	2.5593	2.1925	1.6386	1.0427	2.3965	1.6632	5.2772	3.6783	3.2178	3.5421	3.8399	3.7848	3.0005	4.3294
0.22	0.4300	−0.0323	−0.5883	−0.9692	0.2011	−0.5579	3.2044	1.4385	0.9883	1.4271	1.6349	1.4458	0.7932	2.0115
0.23	−1.5755	−2.2013	−2.6915	−3.1130	−1.9471	−2.4908	1.0298	−0.7383	−1.1490	−0.7774	−0.5461	−0.8400	−1.2988	−0.3248
0.24	−3.3927	−4.0719	−4.4561	−4.9430	−3.7743	−4.2951	−1.0532	−2.5237	−3.0815	−2.6818	−2.5856	−2.7677	−3.1282	−2.2338
0.25	−5.1410	−5.8582	−6.1562	−6.7084	−5.4778	−5.9781	−2.9906	−4.3841	−4.8812	−4.4363	−4.5134	−4.6636	−4.8291	−4.1654
0.26	−6.7028	−7.4217	−7.5659	−8.2543	−6.9447	−7.3340	−4.6446	−5.9917	−6.3977	−6.0862	−6.0945	−6.3345	−6.3415	−5.9272
0.27	−8.1060	−8.7440	−8.8247	−9.3883	−8.1872	−8.6371	−6.1539	−7.3377	−7.7813	−7.4611	−7.5246	−7.7622	−7.7313	−7.3619
0.28	−9.4308	−10.0618	−10.0734	−10.4814	−9.4013	−9.8474	−7.6005	−8.7289	−9.0939	−8.7920	−8.8773	−9.1603	−9.0241	−8.8288
0.29	−10.5393	−11.1304	−11.0829	−11.4828	−10.4654	−10.8252	−8.8542	−9.8390	−10.2159	−10.0130	−9.9946	−10.3412	−10.1753	−10.0420
0.30	−11.5197	−12.0557	−12.0217	−12.3401	−11.4168	−11.7797	−10.0379	−10.8211	−11.2662	−11.0119	−11.0858	−11.3907	−11.2828	−10.9799
0.31	−12.4276	−12.9317	−12.8454	−13.1442	−12.2413	−12.5273	−11.1095	−11.8173	−12.1609	−11.9450	−12.0340	−12.4034	−12.2643	−11.9874
0.32	−13.1572	−13.5257	−13.3978	−13.7140	−12.8621	−13.0696	−11.9939	−12.5192	−12.8999	−12.7198	−12.7691	−13.1876	−13.0484	−12.8375
0.33	−13.8296	−14.0679	−13.8539	−14.1916	−13.4695	−13.6546	−12.8243	−13.2000	−13.5708	−13.3268	−13.4831	−13.8349	−13.7340	−13.5538
0.34	−14.3406	−14.4953	−14.1230	−14.6030	−13.8975	−14.0229	−13.5214	−13.7844	−14.0340	−13.8729	−13.9197	−14.3207	−14.1727	−14.1770
0.35	−14.5470	−14.5914	−14.1781	−14.7470	−14.0830	−14.2091	−14.0271	−14.1316	−14.3557	−14.2004	−14.1699	−14.5892	−14.3925	−14.4972
0.36	−14.5844	−14.6381	−14.2405	−14.6969	−14.2147	−14.3101	−14.4621	−14.4428	−14.5312	−14.3326	−14.4048	−14.7493	−14.5105	−14.6901
0.37	−14.4179	−14.5078	−14.0968	−14.4076	−14.0738	−14.1258	−14.6925	−14.4777	−14.4379	−14.3133	−14.3944	−14.6952	−14.4241	−14.7291
0.38	−14.1370	−14.1243	−13.8027	−13.9065	−13.8475	−13.8117	−14.6622	−14.2696	−14.2433	−14.0602	−14.2411	−14.4195	−14.1716	−14.4826
0.39	−13.8485	−13.7296	−13.4501	−13.4287	−13.7780	−13.3996	−14.4699	−14.0689	−13.9123	−13.7201	−13.9522	−14.0385	−13.7795	−14.1539
0.40	−13.4108	−13.2112	−12.9393	−12.9001	−13.4401	−12.7873	−14.0537	−13.6463	−13.3969	−13.2759	−13.4237	−13.4918	−13.2149	−13.6420
0.41	−12.8457	−12.5340	−12.3927	−12.1717	−12.8061	−12.1068	−13.5315	−13.1215	−12.7578	−12.6833	−12.8352	−12.7506	−12.4988	−12.8958
0.42	−12.1135	−11.6385	−11.6675	−11.0288	−11.9395	−11.1055	−12.9520	−12.4983	−11.8153	−11.9529	−12.0885	−11.7198	−11.4634	−11.9564
0.43	−11.0301	−10.2406	−10.5175	−9.2847	−10.5545	−9.5836	−12.0917	−11.3041	−10.3739	−10.8004	−10.8922	−10.1670	−9.8849	−10.4850
0.44	−9.5532	−8.3835	−8.9507	−7.1051	−8.7744	−7.6217	−10.8090	−9.6128	−8.4383	−9.1383	−9.2107	−8.1262	−7.8192	−8.4506
0.45	−7.6392	−6.1882	−6.9483	−4.6887	−6.7327	−5.1849	−9.0098	−7.4556	−6.0595	−7.1354	−7.0192	−5.7195	−5.3124	−6.0490
0.46	−5.4027	−3.8942	−4.7510	−2.2925	−4.3576	−2.7023	−6.7391	−4.9239	−3.4959	−4.8757	−4.5910	−3.1895	−2.6107	−3.4388
0.47	−3.2150	−1.9201	−2.7452	−0.2548	−1.9743	−0.7200	−4.3031	−2.4895	−1.1295	−2.6682	−2.2772	−0.9511	−0.2487	−1.0755
0.48	−1.3537	−0.4604	−1.0882	1.2331	−0.0636	0.6779	−1.9638	−0.4664	0.6644	−0.8344	−0.3370	0.7829	1.4233	0.6640
0.49	−0.0197	0.3326	−0.0765	1.9724	1.2048	1.3128	−0.1305	0.9604	1.6997	0.5222	0.9591	1.8669	2.3408	1.6887
0.50	0.6677	0.5075	0.2580	2.0366	1.8408	1.3407	0.9929	1.6566	2.0878	1.2477	1.6501	2.2716	2.5009	1.9937
0.51	0.8367	0.3152	0.1343	1.7155	1.8535	1.1203	1.5336	1.8055	1.9503	1.4441	1.8406	2.1861	2.1010	1.7834
0.52	0.5985	−0.1124	−0.2818	1.1456	1.4203	0.7647	1.5087	1.5650	1.4699	1.3166	1.6210	1.7222	1.5094	1.2562
0.53	0.1303	−0.6313	−0.7335	0.5439	0.8309	0.3870	1.1750	1.1395	0.9172	0.9978	1.1871	1.0692	0.8477	0.5620
0.54	−0.3245	−1.0681	−1.1115	−0.0063	0.1972	−0.0103	0.8045	0.7045	0.3472	0.6773	0.6634	0.4405	0.1106	−0.1577
0.55	−0.6910	−1.4494	−1.4329	−0.5581	−0.3517	−0.4505	0.3825	0.1827	−0.2499	0.3279	0.1131	−0.1455	−0.5373	−0.8970
0.56	−0.9896	−1.7407	−1.6249	−0.9836	−0.7490	−0.8670	−0.0263	−0.2929	−0.7310	−0.0752	−0.3886	−0.6692	−1.1158	−1.5602
0.57	−1.1976	−1.8756	−1.6752	−1.2610	−1.0854	−1.3114	−0.4193	−0.6743	−1.1324	−0.4515	−0.8065	−1.1864	−1.7550	−2.0869
0.58	−1.3647	−1.9545	−1.5906	−1.4943	−1.4219	−1.7427	−0.8208	−1.0598	−1.5448	−0.8914	−1.2074	−1.7475	−2.3526	−2.5807
0.59	−1.4384	−2.0300	−1.4279	−1.6775	−1.7623	−2.1186	−1.2239	−1.4209	−1.9299	−1.4040	−1.6931	−2.2437	−2.8396	−2.9521
0.60	−1.4041	−2.0266	−1.3440	−1.9150	−2.0661	−2.5143	−1.7111	−1.8554	−2.2981	−1.9499	−2.1960	−2.6556	−3.1733	−3.0968
0.61	−1.3492	−1.9665	−1.2788	−2.1657	−2.3251	−2.7982	−2.1818	−2.3611	−2.6362	−2.5233	−2.6053	−2.9164	−3.2964	−3.0249
0.62	−1.2609	−1.9140	−1.2218	−2.3148	−2.5498	−3.0312	−2.6226	−2.8212	−2.9062	−2.9745	−2.9410	−2.9787	−3.2550	−2.7111
0.63	−1.2222	−1.8655	−1.2379	−2.4515	−2.6831	−3.2369	−2.9838	−3.1759	−3.0726	−3.2797	−3.0963	−2.8884	−3.0999	−2.2936
0.64	−1.3158	−1.8113	−1.2863	−2.5665	−2.7330	−3.3120	−3.1130	−3.3470	−3.1230	−3.4071	−3.0222	−2.6443	−2.8405	−1.9470
0.65	−1.5280	−1.6588	−1.4295	−2.6633	−2.7229	−3.3558	−3.0649	−3.3440	−3.1239	−3.3022	−2.8818	−2.3568	−2.5823	−1.7194
0.66	−1.8571	−1.5695	−1.6994	−2.8779	−2.6072	−3.3159	−2.8781	−3.2242	−3.0594	−3.0833	−2.6878	−2.1918	−2.4045	−1.5895
0.67	−2.2396	−1.8809	−2.0206	−3.1185	−2.4687	−3.2008	−2.6023	−2.9585	−2.9180	−2.7776	−2.5049	−2.1406	−2.2733	−1.5016
0.68	−2.5877	−2.3709	−2.3691	−3.3168	−2.3928	−3.1196	−2.3447	−2.7025	−2.7616	−2.5334	−2.4303	−2.1539	−2.1533	−1.4824
0.69	−2.8138	−2.7133	−2.6961	−3.5357	−2.3501	−3.0241	−2.1321	−2.5188	−2.6466	−2.4917	−2.4401	−2.1917	−2.0552	−1.5275
0.70	−2.9685	−2.9626	−2.9447	−3.6362	−2.3522	−2.9676	−2.0262	−2.3632	−2.5909	−2.5046	−2.4711	−2.1259	−1.9332	−1.5846
0.71	−3.2304	−3.0116	−3.1274	−3.5612	−2.3492	−2.9356	−1.9933	−2.3150	−2.5793	−2.5404	−2.5056	−1.9918	−1.7936	−1.6428
0.72	−3.4333	−2.9509	−3.1296	−3.3693	−2.3199	−2.8330	−1.9620	−2.2611	−2.5823	−2.5512	−2.4881	−1.8562	−1.7602	−1.6454
0.73	−3.4392	−3.1198	−3.0231	−3.1085	−2.2874	−2.7147	−1.9553	−2.1328	−2.5088	−2.4279	−2.4224	−1.7003	−1.7661	−1.5876
0.74	−3.3273	−3.2600	−3.0058	−2.5753	−2.1907	−2.5562	−1.8805	−2.0171	−2.3369	−2.3162	−2.3092	−1.5387	−1.4640	−1.5654
0.75	−3.0449	−3.1512	−2.9814	−1.8205	−2.0708	−2.3796	−1.7914	−1.8852	−2.1638	−2.2423	−2.1504	−1.4223	−1.1909	−1.5505
0.76	−2.7082	−3.0758	−2.8684	−1.5481	−2.0045	−2.2545	−1.7358	−1.7201	−1.9901	−2.1220	−2.0496	−1.3304	−1.4245	−1.4687
0.77	−2.5245	−3.0472	−2.6986	−1.7668	−1.8476	−2.0923	−1.6282	−1.6145	−1.8138	−2.0424	−1.9681	−1.2928	−1.7178	−1.3945
0.78	−2.3700	−2.9247	−2.4498	−2.0337	−1.6652	−1.9292	−1.5386	−1.5121	−1.6768	−1.9553	−1.8850	−1.3613	−1.8804	−1.3908
0.79	−2.1836	−2.7952	−2.2168	−2.3743	−1.6457	−1.8377	−1.5581	−1.4660	−1.5704	−1.8425	−1.8794	−1.4436	−2.0532	−1.4393
0.80	−2.0526	−2.6406	−2.1312	−2.3773	−1.6643	−1.8020	−1.6210	−1.5108	−1.5028	−1.7435	−1.8737	−1.5182	−1.8096	−1.4778
0.81	−1.9809	−2.4471	−2.1086	−2.0006	−1.7231	−1.8703	−1.6795	−1.5905	−1.4979	−1.6326	−1.8689	−1.6918	−1.4664	−1.5507
0.82	−1.9246	−2.2852	−2.1256	−1.9560	−1.8530	−1.9456	−1.7023	−1.7434	−1.5317	−1.6559	−1.8896	−1.8638	−1.5435	−1.6715
0.83	−1.8900	−2.1526	−2.1747	−2.0227	−1.8602	−1.9381	−1.7025	−1.8891	−1.5561	−1.8345	−1.8590	−1.9526	−1.5767	−1.8218
0.84	−1.9113	−2.0537	−2.2965	−1.9196	−1.7826	−1.9620	−1.7442	−1.9696	−1.5407	−1.9365	−1.8990	−2.0199	−1.5450	−2.0060
0.85	−1.9911	−2.0360	−2.4771	−1.9145	−1.7811	−2.0019	−1.8875	−2.0642	−1.5820	−1.9270	−2.0148	−1.9990	−1.6569	−2.1361
0.86	−2.1012	−2.0623	−2.5879	−1.8938	−1.9250	−2.0153	−2.0510	−2.0667	−1.7908	−1.9167	−2.0780	−1.9026	−1.7782	−2.1361
0.87	−2.2421	−2.1319	−2.7133	−1.9511	−2.1811	−2.0648	−2.1117	−2.0298	−2.0483	−1.8995	−2.1293	−1.9301	−1.8639	−2.1432
0.88	−2.3820	−2.2239	−2.8869	−2.0721	−2.3306	−2.0814	−2.1188	−2.0587	−2.2263	−1.9662	−2.1274	−1.9967	−1.9220	−2.2337
0.89	−2.4926	−2.3198	−2.9723	−2.0656	−2.3612	−2.0662	−2.1449	−2.0355	−2.3018	−2.0885	−2.0752	−2.0227	−1.9416	−2.3037
0.90	−2.5867	−2.4120	−3.0267	−2.1226	−2.3470	−2.0741	−2.2245	−2.0425	−2.2386	−2.1511	−2.1131	−2.0658	−1.9675	−2.3131
0.91	−2.6910	−2.4719	−3.1898	−2.1939	−2.3154	−2.0315	−2.4313	−2.1044	−2.1440	−2.1610	−2.1997	−2.1234	−2.0051	−2.3130
0.92	−2.1239	−2.2543	−2.2711	−2.0503	−1.9348	−2.0259	−1.8800	−1.9112	−1.8927	−1.8921	−1.9187	−1.7769	−1.7492	−1.7491
0.93	−1.5368	−2.0359	−1.3407	−1.9229	−1.6527	−2.0330	−1.3000	−1.7140	−1.6796	−1.6716	−1.6491	−1.4032	−1.5061	−1.1764
0.94	−0.7921	−1.2895	−1.2721	−2.6260	0.5191	−2.8470	1.6742	0.5498	−0.0008	−0.6604	−0.7771	0.9204	0.6572	0.7244
0.95	−1.5758	−2.1094	−1.3804	−1.8510	−1.8914	−1.8825	−1.6178	−1.5831	−1.7069	−1.8067	−1.7338	−1.2666	−1.4849	−1.0078

**Table A2:** Accelerations measured for tests from 15 to 28.

Time in ms	3 182 HB							4 195 HB						
	Test 15	Test 16	Test 17	Test 18	Test 19	Test 20	Test 21	Test 22	Test 23	Test 24	Test 25	Test 26	Test 27	Test 28
0.01	1.0241	0.6706	1.1855	0.2205	0.3973	1.4264	0.3206	1.0076	0.8720	0.4529	0.4140	0.6482	0.3978	0.9311
0.02	2.2977	1.9553	2.4047	1.4995	1.7847	2.5947	1.5576	2.2042	2.0833	1.7314	1.6128	1.8970	1.7056	2.0816
0.03	3.7904	3.4366	3.8641	2.8606	3.1753	4.0711	2.9386	3.5765	3.4379	3.1192	2.9144	3.2380	3.0284	3.4279
0.04	5.4545	5.0924	5.5458	4.3733	4.6482	5.7239	4.5409	5.0956	4.9668	4.5995	4.3622	4.6936	4.4007	4.9558
0.05	6.8893	6.4856	6.9800	5.9314	6.2235	7.1600	6.1283	6.3705	6.2793	6.0889	5.8567	6.0575	5.8848	6.3129
0.06	8.2741	7.9314	8.3636	7.4058	7.6714	8.5827	7.6411	7.7116	7.5823	7.5373	7.2884	7.4195	7.2409	7.6799
0.07	9.6664	9.4431	9.7985	8.9399	9.1222	9.9085	9.1652	9.1457	8.9921	8.9683	8.6950	8.8453	8.5914	9.1440
0.08	10.7088	10.5453	10.8420	10.2980	10.4819	10.9572	10.4275	10.2038	10.0584	10.2165	9.9735	9.9741	9.8639	10.2728
0.09	11.6724	11.6038	11.8549	11.4179	11.5287	12.0459	11.5720	11.2385	11.0858	11.2827	11.0851	11.0120	10.8651	11.2987
0.10	12.3896	12.4433	12.6386	12.5082	12.4850	12.7578	12.6323	12.0223	11.9497	12.2094	12.0585	11.9485	11.8109	12.1338
0.11	12.7888	12.9033	13.0057	13.2077	13.0965	13.1954	13.3329	12.4408	12.4573	12.8006	12.6843	12.5141	12.4435	12.5742
0.12	13.1480	13.2763	13.3512	13.7059	13.4375	13.6396	13.8662	12.8335	12.8832	13.2149	13.1278	12.9612	12.7980	12.9618
0.13	13.0642	13.3181	13.2368	14.1432	13.7371	13.5637	14.1728	12.8896	12.9834	13.5163	13.4107	13.1963	13.0599	13.1270
0.14	12.5683	12.8985	12.6374	14.0152	13.4958	13.0993	13.9871	12.5168	12.6016	13.2582	13.1932	12.8319	12.8483	12.8061
0.15	11.7492	12.0906	11.7985	13.4797	12.8071	12.2927	13.4449	11.8192	11.9448	12.5438	12.6800	12.1563	12.2629	12.1311
0.16	10.4373	10.8242	10.3998	12.5406	11.7857	10.9163	12.3889	10.6689	10.8699	11.5162	11.7385	11.1215	11.3682	10.9785
0.17	8.9711	9.2705	8.7530	11.0360	10.2098	9.4183	10.8435	9.2328	9.4153	10.0043	10.2961	9.6744	9.9718	9.4565
0.18	7.3222	7.6177	7.0942	9.4320	8.5480	7.7963	9.2312	7.6714	7.8885	8.3783	8.7850	8.2113	8.4499	7.8671
0.19	5.4168	5.7248	5.0969	7.6529	6.7974	5.8618	7.4293	5.8683	6.0798	6.6162	7.0158	6.4768	6.7785	6.0684
0.22	3.4637	3.6340	3.0365	5.5332	4.6937	3.8105	5.3607	3.9497	4.0022	4.5537	4.9736	4.4521	4.8244	4.1051
0.21	1.3805	1.5359	0.9903	3.3818	2.6169	1.6024	3.2472	1.9627	1.9565	2.4704	2.9525	2.4772	2.8707	2.1008
0.22	−0.8081	−0.6435	−1.2471	1.0990	0.5028	−0.6378	0.9659	−0.1239	−0.1740	0.3343	0.7561	0.3760	0.8083	−0.0668
0.23	−2.9356	−2.7992	−3.3606	−1.2426	−1.7023	−2.7449	−1.3629	−2.1777	−2.3088	−1.8698	−1.4615	−1.7504	−1.3691	−2.2483
0.24	−5.0143	−4.7895	−5.3478	−3.3427	−3.6833	−4.7803	−3.5245	−4.1903	−4.2319	−3.9181	−3.5420	−3.7060	−3.3779	−4.2825
0.25	−6.8872	−6.6925	−7.2063	−5.3627	−5.5781	−6.6325	−5.5859	−6.0961	−6.0668	−5.8852	−5.5600	−5.6735	−5.3452	−6.2087
0.26	−8.4975	−8.4123	−8.7179	−7.2332	−7.3371	−8.2647	−7.4398	−7.7467	−7.7499	−7.6792	−7.3314	−7.4065	−7.1584	−7.9166
0.27	−9.9570	−9.8796	−10.1076	−8.8037	−8.7999	−9.7606	−9.0459	−9.2091	−9.2133	−9.1575	−8.8755	−8.8484	−8.6692	−9.4365
0.28	−11.2125	−11.1803	−11.3601	−10.2482	−10.1894	−11.0625	−10.5179	−10.5021	−10.5495	−10.5446	−10.2948	−10.1983	−10.0920	−10.8030
0.29	−12.2912	−12.2941	−12.3502	−11.5255	−11.4444	−12.1990	−11.7601	−11.6534	−11.7061	−11.7669	−11.4845	−11.3392	−11.3256	−11.9675
0.30	−13.2574	−13.2548	−13.2851	−12.5975	−12.4585	−13.1682	−12.7717	−12.6919	−12.7246	−12.7186	−12.5548	−12.3103	−12.3292	−12.9301
0.31	−14.0197	−14.0147	−13.9890	−13.5836	−13.3535	−13.8843	−13.6488	−13.4695	−13.6003	−13.5383	−13.4939	−13.1671	−13.2191	−13.6772
0.32	−14.5538	−14.5595	−14.4097	−14.3272	−13.9592	−14.3976	−14.2559	−14.0418	−14.2819	−14.1040	−14.1151	−13.7207	−13.8418	−14.2109
0.33	−14.9085	−14.9402	−14.7495	−14.7490	−14.2618	−14.7193	−14.6814	−14.4017	−14.6879	−14.4041	−14.5104	−14.0376	−14.2291	−14.5079
0.34	−15.0106	−15.0503	−14.7704	−14.9648	−14.4330	−14.8378	−14.9872	−14.5205	−14.7093	−14.5200	−14.6655	−14.1554	−14.4323	−14.5582
0.35	−14.9091	−14.9312	−14.5298	−14.9346	−14.3518	−14.7371	−14.9945	−14.4925	−14.4640	−14.3931	−14.5510	−14.0159	−14.3904	−14.4044
0.36	−14.6871	−14.5999	−14.1931	−14.7106	−14.0851	−14.3525	−14.7674	−14.3095	−14.0163	−14.0805	−14.2948	−13.7011	−14.1645	−14.0647
0.37	−14.2676	−13.9874	−13.5535	−14.3697	−13.7082	−13.7155	−14.3934	−13.9236	−13.3122	−13.5779	−13.8460	−13.2172	−13.7539	−13.4207
0.38	−13.4515	−12.9638	−12.4821	−13.7322	−12.9558	−12.6294	−13.6578	−13.0170	−12.1427	−12.5989	−12.9660	−12.3012	−12.9524	−12.2434
0.39	−11.9797	−11.2954	−10.8209	−12.5349	−11.6208	−10.8815	−12.3704	−11.0963	−10.2355	−10.9684	−11.4245	−10.7423	−11.5645	−10.3696
0.40	−9.7880	−8.9718	−8.4619	−10.6707	−9.6460	−8.4534	−10.4612	−8.2280	−7.6187	−8.6956	−9.1332	−8.6015	−9.5212	−7.8789
0.41	−6.9809	−6.1630	−5.6361	−8.1014	−7.0619	−5.4515	−7.9499	−4.8619	−4.5660	−5.9393	−6.2616	−5.9435	−7.0104	−4.9859
0.42	−3.8633	−3.1246	−2.7351	−4.9658	−4.1996	−2.2803	−5.0426	−1.5384	−1.4857	−2.9906	−3.1929	−3.1477	−4.3205	−2.0521
0.43	−0.9933	−0.3846	−0.2298	−1.7445	−1.4607	0.4746	−2.1242	1.0895	1.0989	−0.2762	−0.4497	−0.7346	−1.7374	0.4070
0.44	1.2761	1.7215	1.5922	0.9382	0.7679	2.5271	0.4118	2.8500	2.9079	1.8294	1.6342	1.1588	0.5168	2.1955
0.45	2.8046	3.1079	2.6740	2.8350	2.2910	3.7249	2.3357	3.8782	3.8120	3.1558	2.9737	2.4664	2.3135	3.2196
0.46	3.4633	3.6364	2.9963	3.8381	3.1096	4.0690	3.4538	4.1651	3.8821	3.6428	3.5959	3.0866	3.3620	3.4632
0.47	3.4779	3.5595	2.8690	4.0629	3.2790	3.8944	3.7588	3.8819	3.5015	3.5332	3.7196	3.2300	4.0745	3.2422
0.48	3.1329	3.1227	2.5635	3.9620	3.1483	3.3670	3.7527	3.2703	2.9244	3.1593	3.4599	3.0132	4.4904	2.8411
0.49	2.4612	2.3953	2.0952	3.5203	2.8818	2.6181	3.9307	2.5782	2.3599	2.6474	2.8148	2.4712	3.7031	2.3167
0.50	1.7220	1.6426	1.5833	2.6170	2.3184	1.9134	3.5776	2.0003	1.9233	2.0705	2.0666	1.8828	2.3575	1.8430
0.51	1.0685	1.0023	1.0982	1.6614	1.6433	1.3426	2.4312	1.4794	1.5128	1.5143	1.3762	1.3425	1.3140	1.4889
0.52	0.5545	0.4984	0.6711	0.8940	1.0874	1.0010	1.3238	1.1375	1.0764	1.0519	0.8952	0.9327	0.3385	1.1511
0.53	0.3689	0.3198	0.4593	0.4948	0.7026	0.9161	0.4150	0.9945	0.6330	0.8105	0.7512	0.6957	0.1610	0.8992
0.54	0.3701	0.3438	0.4684	0.6097	0.6506	0.8690	−0.1442	0.7754	0.1520	0.7198	0.7276	0.5340	0.6441	0.7056
0.55	0.3724	0.3897	0.5578	0.8129	0.7451	0.7867	0.0879	0.5389	−0.2266	0.6581	0.6496	0.3953	0.4737	0.5525
0.56	0.3999	0.4193	0.5436	0.8420	0.7489	0.6725	0.5285	0.2682	−0.5217	0.5800	0.5339	0.2264	0.2133	0.4695
0.57	0.2762	0.2579	0.3414	0.6844	0.7035	0.3303	0.7447	−0.2142	−0.8400	0.3716	0.2894	−0.0227	0.3768	0.3791
0.58	−0.0013	−0.1196	−0.0070	0.3786	0.5969	−0.1329	1.0817	−0.6598	−1.1420	0.0232	−0.0265	−0.3189	0.5683	0.2577
0.59	−0.3200	−0.6048	−0.4719	0.0380	0.3648	−0.6585	1.2583	−1.0586	−1.4309	−0.3736	−0.3437	−0.6952	0.8638	0.0483
0.60	−0.7678	−1.1576	−0.9408	−0.4773	0.0047	−1.2296	0.9229	−1.5217	−1.7106	−0.8129	−0.7391	−1.1187	1.0501	−0.2620
0.61	−1.2624	−1.6441	−1.3537	−1.1086	−0.5171	−1.6621	0.3495	−1.8915	−1.9476	−1.3017	−1.1287	−1.4763	0.9823	−0.5168
0.62	−1.7866	−2.0458	−1.8405	−1.6689	−1.0997	−2.0458	−0.1309	−2.2105	−2.1560	−1.7323	−1.5098	−1.8184	0.6963	−0.7695
0.63	−2.3662	−2.4333	−2.3228	−2.2928	−1.6290	−2.4527	−0.6028	−2.4289	−2.2536	−2.1038	−1.8777	−2.0681	0.1204	−1.0817
0.64	−2.8126	−2.7945	−2.7158	−2.7788	−2.0277	−2.7637	−1.1690	−2.5112	−2.2106	−2.4752	−2.1526	−2.1640	−0.4913	−1.4241
0.65	−3.1126	−3.1147	−3.1084	−3.0169	−2.3647	−2.9696	−1.7628	−2.5769	−2.0920	−2.7987	−2.3459	−2.1704	−1.1612	−1.8744
0.66	−3.2951	−3.2709	−3.3439	−3.1762	−2.6723	−2.9586	−2.3031	−2.5698	−1.8604	−2.9198	−2.4431	−2.0924	−2.0180	−2.3006
0.67	−3.3421	−3.2242	−3.3646	−3.0784	−2.7950	−2.7490	−2.7532	−2.4110	−1.6114	−2.8016	−2.4021	−1.9657	−2.7354	−2.5757
0.68	−3.3129	−3.0135	−3.2965	−2.7851	−2.7896	−2.4751	−3.0918	−2.1140	−1.3743	−2.4766	−2.2411	−1.8433	−3.2779	−2.7380
0.69	−3.1039	−2.6083	−3.0053	−2.4853	−2.6596	−2.0632	−3.2328	−1.6332	−1.0548	−1.9909	−1.9618	−1.6700	−3.6594	−2.7403
0.70	−2.6855	−2.1838	−2.5416	−2.0758	−2.3379	−1.5990	−3.1682	−1.1598	−0.5558	−1.5436	−1.6854	−1.4296	−3.6246	−2.6226
0.71	−2.2242	−1.7318	−2.0485	−1.6367	−2.0341	−0.8936	−2.9390	−0.9010	−0.1942	−1.2590	−1.4605	−1.2008	−3.3703	−2.4136
0.72	−1.7814	−1.0786	−1.5225	−1.2016	−1.7730	−0.2984	−2.5250	−0.6875	−0.2735	−1.0257	−1.1905	−0.9599	−2.9640	−2.0855
0.73	−1.4687	−0.7549	−1.0414	−0.6626	−1.4397	−0.3834	−1.9909	−0.5811	−0.5518	−0.8113	−1.0090	−0.7521	−2.4117	−1.7288
0.74	−1.2936	−0.8873	−0.7513	−0.0752	−1.1878	−0.6331	−1.3403	−0.7314	−0.8434	−0.6672	−0.9104	−0.6782	−2.0136	−1.4291
0.75	−1.0529	−0.9791	−0.7756	0.1913	−1.0204	−0.8074	−0.6387	−0.8592	−1.0944	−0.4613	−0.7985	−0.7062	−1.6203	−1.1963
0.76	−0.9076	−1.1389	−1.0224	−0.1020	−0.8879	−0.9670	−0.2839	−0.8691	−1.0515	−0.3059	−0.7927	−0.7582	−1.1810	−1.0266
0.77	−0.9246	−1.2726	−1.1887	−0.5570	−0.8573	−0.7240	−0.4359	−0.9238	−0.9179	−0.4095	−0.8131	−0.8210	−0.9058	−0.9020
0.78	−0.8949	−1.0855	−1.2327	−0.9056	−0.7420	−0.5313	−0.7910	−0.8362	−1.0092	−0.6415	−0.7563	−0.8446	−0.6498	−0.8030
0.79	−0.9579	−1.0806	−1.1994	−1.0730	−0.5451	−0.7536	−1.0642	−0.7231	−1.0237	−0.8337	−0.7344	−0.8120	−0.4396	−0.7434
0.80	−1.0830	−1.2774	−1.0829	−0.9091	−0.5898	−0.8687	−1.0903	−0.7975	−0.9207	−0.9383	−0.7221	−0.8310	−0.3956	−0.7129
0.81	−1.0857	−1.3056	−1.1167	−0.8210	−0.7544	−0.9170	−0.9039	−0.8094	−0.8935	−0.9243	−0.7047	−0.9126	−0.4036	−0.6964
0.82	−1.1494	−1.3876	−1.2538	−1.0627	−0.9081	−0.9889	−0.7885	−0.7952	−0.8633	−0.9230	−0.7985	−0.9437	−0.5506	−0.6937
0.83	−1.2701	−1.4657	−1.2309	−1.1534	−1.0893	−1.0109	−0.8548	−0.8805	−0.8094	−1.0226	−0.9238	−0.9232	−0.7637	−0.6998
0.84	−1.3138	−1.4368	−1.2240	−1.1064	−1.1143	−1.0540	−0.9228	−0.9530	−0.7891	−1.1360	−1.0217	−0.9350	−0.9308	−0.7722
0.85	−1.4221	−1.4169	−1.3022	−1.1312	−1.0050	−1.0597	−0.8866	−0.9900	−0.7377	−1.1526	−1.0585	−0.9155	−1.1120	−0.9444
0.86	−1.5215	−1.3367	−1.2538	−1.0406	−1.0014	−1.0355	−0.8515	−0.9854	−0.7296	−1.0912	−0.9868	−0.8724	−1.2032	−1.0692
0.87	−1.5374	−1.2553	−1.2545	−0.8965	−1.0130	−1.0291	−0.9081	−0.9533	−0.7522	−1.0227	−0.8940	−0.8936	−1.2586	−1.1161
0.88	−1.5483	−1.2597	−1.3109	−0.8504	−0.9822	−0.9849	−0.9355	−0.9995	−0.7448	−0.9519	−0.8538	−0.9109	−1.3412	−1.1288
0.89	−1.4805	−1.2065	−1.2474	−0.7803	−0.9816	−0.9634	−0.9220	−1.0506	−0.7908	−0.8567	−0.8256	−0.9162	−1.3048	−1.0933
0.90	−1.3793	−1.1806	−1.2471	−0.7402	−0.9828	−0.9829	−0.9451	−1.0696	−0.8449	−0.7475	−0.8370	−0.9451	−1.2369	−1.0774
0.91	−1.3388	−1.1955	−1.2408	−0.7792	−1.0074	−0.9760	−0.9220	−1.1184	−0.9317	−0.6140	−0.8332	−0.9489	−1.2268	−1.1080
0.92	−1.2346	−1.1288	−1.1406	−0.8329	−0.8221	−0.7993	−0.7676	−0.8144	−0.7636	−0.7929	−0.7787	−0.7977	−0.8343	−0.8301
0.93	−1.1131	−1.0759	−1.0677	−0.8417	−0.6297	−0.6391	−0.6672	−0.5038	−0.5968	−0.9846	−0.7470	−0.6557	−0.4412	−0.5193
0.94	−2.4231	−3.3709	−3.3569	−2.8063	−3.2125	−2.6181	−2.8568	−2.7464	−1.2112	−2.5890	−1.9694	−2.3275	−4.1899	−3.3133
0.95	−0.9386	−1.0114	−0.7852	−0.6483	−0.5878	−0.5889	−0.5980	−0.5086	−0.8834	−0.7230	−0.6458	−0.5804	−0.3247	−0.4183

**Table A3:** Accelerations measured for tests from 29 to 42.

Time in ms	5 197 HB							6 271 HB						
	Test 29	Test 30	Test 31	Test 32	Test 33	Test 34	Test 35	Test 36	Test 37	Test 38	Test 39	Test 40	Test 41	Test 42
0.01	−1.1294	0.1727	0.3115	1.2366	0.6164	0.1690	0.6784	0.6217	0.8492	0.8258	0.6108	0.4785	0.7953	1.4463
0.02	−1.1038	1.3373	1.5678	2.4553	1.7463	1.1842	1.8125	1.7094	1.8773	1.9867	1.5164	1.5319	1.9428	2.5517
0.03	−0.6581	2.5633	2.8607	3.7877	3.0288	2.4530	3.0687	3.0019	3.1037	3.2994	2.6733	2.6804	3.2090	3.7786
0.04	0.1587	3.9078	4.2482	5.2974	4.4605	3.9586	4.4607	4.4362	4.5334	4.7595	4.0372	3.9159	4.5611	5.1321
0.05	1.2163	5.3719	5.7597	6.6693	5.8284	5.4383	5.9353	5.8856	5.7993	6.1615	5.4027	5.2144	5.8158	6.3942
0.06	2.5163	6.7862	7.1328	7.9894	7.2787	6.9207	7.3703	7.3169	7.0856	7.5825	6.7978	6.5291	7.1146	7.6050
0.07	4.0121	8.1441	8.4749	9.3437	8.7075	8.4267	8.6936	8.6186	8.4236	8.9703	8.1883	7.8349	8.3578	8.7476
0.08	5.4994	9.4191	9.7280	10.3932	9.7855	9.6717	9.8608	9.7215	9.4135	10.0054	9.3332	8.9690	9.3868	9.7012
0.09	7.0590	10.4685	10.7253	11.3713	10.8278	10.8472	10.8879	10.6897	10.3407	10.9155	10.3675	9.9209	10.3566	10.5801
0.10	8.5796	11.3826	11.6899	12.1883	11.7403	11.8956	11.7029	11.4890	11.1331	11.7249	11.2135	10.7364	11.1351	11.2093
0.11	9.8369	12.0645	12.3245	12.6849	12.3278	12.6145	12.3118	12.0055	11.6060	12.1977	11.7134	11.2680	11.6826	11.6530
0.12	10.9754	12.5439	12.7116	13.1348	12.7873	13.2142	12.8092	12.3484	12.0401	12.5819	12.1661	11.6840	12.0718	12.0187
0.13	11.9028	12.9177	13.0738	13.2068	13.0134	13.5986	13.0117	12.5834	12.2564	12.8488	12.4243	12.0442	12.2919	12.0430
0.14	12.5803	12.9239	12.9410	12.7562	12.7921	13.5972	12.8695	12.4953	12.0530	12.5966	12.3740	12.0398	12.1132	11.7562
0.15	13.1454	12.5506	12.4129	11.9936	12.2756	13.1960	12.3392	11.9426	11.5298	11.9331	11.9836	11.7020	11.5188	11.0866
0.16	13.4768	11.7866	11.5675	10.7462	11.3996	12.3090	11.2914	10.9637	10.5997	10.8619	11.0458	10.9348	10.5814	9.9315
0.17	13.4323	10.6126	10.2030	9.2494	10.1296	10.9807	9.9780	9.6178	9.2852	9.3098	9.7408	9.7239	9.2337	8.5796
0.18	13.0249	9.2250	8.7096	7.7039	8.6729	9.3808	8.4563	7.9786	7.7233	7.6100	8.1517	8.3646	7.6391	6.9537
0.19	12.1829	7.6506	7.0103	5.8304	6.9794	7.6176	6.7428	6.1039	5.9766	5.8024	6.3632	6.7257	5.8868	5.1375
0.22	10.8599	5.9031	4.9749	3.7958	5.0560	5.7204	4.9306	4.1240	4.0979	3.7898	4.5704	4.8948	3.9574	3.3070
0.21	9.2654	3.9977	3.0238	1.7324	3.0300	3.6461	2.9223	2.0484	2.1653	1.7069	2.6389	2.9579	1.9416	1.2216
0.22	7.5577	1.9501	1.0004	−0.4321	0.9033	1.5010	0.8744	−0.1375	0.1523	−0.4610	0.5648	0.7444	−0.0996	−0.8568
0.23	5.6941	−0.1238	−1.1517	−2.4874	−1.2310	−0.6577	−1.1666	−2.2189	−1.9577	−2.7079	−1.5262	−1.3914	−2.1999	−2.8162
0.24	3.6733	−2.2669	−3.1159	−4.4641	−3.2743	−2.8177	−3.2652	−4.3048	−4.1171	−4.9122	−3.7156	−3.3959	−4.2898	−4.8633
0.25	1.5655	−4.3557	−5.1242	−6.3922	−5.2478	−4.8411	−5.1803	−6.4820	−6.2049	−6.9777	−5.7906	−5.4511	−6.2835	−6.7563
0.26	−0.6402	−6.2194	−6.9907	−8.0747	−7.0379	−6.6940	−6.9455	−8.3630	−8.0979	−8.8317	−7.6063	−7.2783	−8.1014	−8.5212
0.27	−2.8702	−7.9260	−8.4500	−9.5564	−8.6175	−8.4112	−8.5591	−9.9887	−9.7732	−10.5088	−9.3066	−8.9709	−9.7262	−10.1795
0.28	−4.9071	−9.4138	−9.8508	−10.8597	−10.0382	−9.9137	−9.8707	−11.4881	−11.2225	−11.9489	−10.7805	−10.5664	−11.1305	−11.4869
0.29	−6.7514	−10.6922	−11.1121	−11.9057	−11.2837	−11.2557	−11.0785	−12.6802	−12.4798	−13.1143	−12.0454	−11.8679	−12.3552	−12.5735
0.30	−8.4518	−11.8823	−12.1125	−12.8164	−12.3472	−12.3920	−12.1254	−13.6832	−13.5248	−14.0578	−13.1817	−12.9779	−13.3330	−13.4860
0.31	−9.9207	−12.8562	−13.0665	−13.5244	−13.2258	−13.2975	−12.9672	−14.4828	−14.2992	−14.6946	−14.0031	−13.8473	−13.9156	−14.1568
0.32	−11.2144	−13.5828	−13.7453	−14.0384	−13.8573	−14.0906	−13.7193	−14.9559	−14.8458	−15.0597	−14.5898	−14.4255	−14.2505	−14.6786
0.33	−12.3812	−14.1297	−14.1395	−14.4629	−14.2551	−14.6368	−14.1598	−15.2281	−15.1503	−15.2714	−15.0069	−14.7483	−14.3288	−14.9248
0.34	−13.3012	−14.4204	−14.3951	−14.6804	−14.4366	−14.9434	−14.2887	−15.2810	−15.1746	−15.2420	−15.0826	−14.7614	−14.1514	−14.9292
0.35	−14.0658	−14.4963	−14.3514	−14.6214	−14.4374	−15.0612	−14.2496	−15.0009	−14.9846	−14.8830	−14.9376	−14.6347	−13.8309	−14.6808
0.36	−14.6249	−14.3896	−14.1588	−14.3083	−14.2767	−14.8744	−13.9434	−14.1910	−14.3700	−13.9113	−14.4202	−14.1651	−13.0084	−13.6653
0.37	−14.8613	−14.0586	−13.8459	−13.5997	−13.8967	−14.4957	−13.5269	−12.4828	−12.9563	−12.0844	−13.1692	−12.8745	−11.3657	−11.7995
0.38	−14.9268	−13.5007	−13.1393	−12.3890	−13.1549	−13.8075	−12.7886	−10.0657	−10.6921	−9.5256	−11.2428	−10.8213	−9.1530	−9.2167
0.39	−14.7879	−12.4248	−11.8840	−10.6010	−11.8153	−12.3863	−11.2983	−7.1891	−7.6555	−6.4193	−8.6321	−8.0106	−6.5054	−6.0310
0.40	−14.4118	−10.6287	−9.9919	−8.2053	−9.8285	−10.2812	−9.1664	−4.0090	−4.1769	−3.1488	−5.5269	−4.6658	−3.7470	−2.8589
0.41	−13.8210	−8.2615	−7.4842	−5.4458	−7.3125	−7.5653	−6.4773	−0.9943	−0.9463	−0.2931	−2.6092	−1.5373	−1.4278	−0.1671
0.42	−12.6406	−5.4727	−4.7468	−2.7538	−4.5177	−4.3916	−3.5418	1.5665	1.5950	1.9731	−0.0924	1.0623	0.3864	2.0029
0.43	−10.7762	−2.6586	−2.2531	−0.5036	−1.9529	−1.3254	−1.0023	3.4312	3.2904	3.4686	2.1674	2.9328	1.6649	3.3677
0.44	−8.4751	−0.1839	−0.1064	1.0767	0.1211	1.2660	0.9850	4.2548	4.0438	3.9352	3.7059	3.7574	2.2985	3.8345
0.45	−5.7903	1.8957	1.7064	2.0432	1.6367	3.0916	2.2617	4.1775	4.4353	4.3004	5.3213	4.0921	2.5428	4.5756
0.46	−3.1348	3.3337	3.0277	2.8446	2.5277	3.9462	2.7374	4.0286	4.7875	4.7328	6.0185	4.5143	2.5758	4.8495
0.47	−0.9222	4.0313	3.7886	3.5474	2.9584	4.0313	2.7586	3.7609	4.1828	3.9754	4.2140	4.1590	2.4140	3.6190
0.48	0.9472	4.1231	3.9118	3.7025	3.0847	3.6905	2.4302	2.8204	2.7168	2.5828	2.1094	2.9501	2.0875	2.2895
0.49	2.4417	3.6395	3.9710	3.9532	2.8567	3.2073	1.9511	1.6712	1.3563	1.4662	0.9519	1.8069	1.5377	1.1901
0.50	3.4300	2.8118	3.5563	3.5108	2.6698	2.6836	1.5638	0.8003	0.3376	0.4742	0.2535	0.8923	0.9702	0.2538
0.51	3.9125	1.9739	2.2223	1.5174	2.7227	2.0354	1.1299	0.2541	0.1619	0.4147	0.9154	0.5929	0.6867	0.5981
0.52	3.7802	1.2311	1.0051	0.0194	2.2121	1.2518	0.7836	0.5251	0.7993	1.0885	1.6409	1.1134	0.7335	1.1867
0.53	3.2132	0.7474	0.1632	−0.4367	1.1991	0.5049	0.6585	1.2144	1.1357	1.1660	1.0563	1.4285	1.0631	1.0061
0.54	2.3422	0.5417	−0.4024	−0.5516	0.3809	0.0940	0.5111	1.3396	1.1261	1.1251	1.0543	1.2670	1.3727	1.1703
0.55	1.4048	0.4262	0.0364	−0.0348	−0.2757	0.0856	0.3698	1.1604	1.3047	1.3745	1.5582	1.2178	1.3237	1.4501
0.56	0.6945	0.3742	0.4889	0.4284	−0.4035	0.1935	0.2296	0.9422	1.4967	1.5096	1.6137	1.1892	1.0495	1.4704
0.57	0.1120	0.3992	0.1358	0.5114	0.3189	0.2246	−0.0657	0.5389	1.5031	1.5278	1.3360	0.9061	0.7610	1.3360
0.58	−0.1230	0.4009	0.3836	1.0911	0.9606	0.1090	−0.3754	0.3930	1.3357	1.4299	0.9238	0.4531	0.5436	1.0570
0.59	0.1552	0.3471	1.1363	1.7386	1.1846	−0.1531	−0.6352	0.7463	1.0942	1.2673	0.7099	0.3785	0.4762	0.7784
0.60	0.4573	0.2301	1.4822	1.8288	1.3644	−0.3965	−0.8658	1.0888	0.8156	0.8474	0.8027	0.6156	0.3557	0.3942
0.61	0.6651	−0.0376	1.4630	1.0983	1.2402	−0.6699	−1.0593	1.0614	0.3435	0.1840	0.8441	0.6616	−0.0277	0.0322
0.62	0.7530	−0.4071	1.1263	0.3293	0.7597	−1.1920	−1.3225	0.6324	−0.1454	0.0160	0.4798	0.5450	−0.5733	0.0872
0.63	0.6756	−0.8608	0.3080	0.2411	0.3116	−1.8230	−1.6407	−0.1580	−0.2138	0.1624	−0.1779	0.1185	−1.0287	0.2419
0.64	0.7969	−1.3935	−0.3816	−0.0200	−0.0413	−2.3806	−1.8887	−1.0405	−0.0858	0.0197	−0.3579	−0.7280	−1.2017	0.1787
0.65	1.0020	−1.9286	−0.9273	−0.8292	−0.5560	−2.7642	−2.0556	−1.7532	−0.1932	−0.2768	0.0596	−1.3553	−1.1963	−0.0736
0.66	1.0661	−2.3496	−1.7718	−1.8788	−1.1309	−2.8375	−2.0629	−2.2562	−0.4739	−1.0452	0.3329	−1.7136	−1.1446	−0.6501
0.67	0.8932	−2.6578	−2.5326	−2.8088	−1.6209	−2.7337	−1.9713	−2.5048	−1.0634	−2.0634	0.2301	−2.2053	−0.9855	−1.3713
0.68	0.2666	−2.8981	−3.0208	−3.2815	−2.0773	−2.6112	−1.8468	−2.6009	−1.8684	−2.4316	−0.3552	−2.4969	−1.0307	−1.7553
0.69	−0.5637	−2.9621	−3.3212	−3.1953	−2.4030	−2.4328	−1.6568	−2.5663	−2.2241	−2.5751	−1.2460	−2.3762	−1.2306	−1.9756
0.70	−1.2877	−2.8161	−3.3290	−2.9747	−2.6510	−2.2148	−1.4688	−2.3224	−2.3669	−2.7852	−1.6280	−2.1144	−1.1262	−2.2547
0.71	−1.9832	−2.5563	−3.1678	−2.9398	−2.8306	−1.8783	−1.1209	−1.9921	−2.5753	−2.6137	−1.6718	−1.6597	−0.8860	−2.3360
0.72	−2.5576	−2.1261	−2.8400	−2.9102	−2.7760	−1.3778	−0.7029	−1.5505	−2.4977	−2.2466	−1.9069	−1.1301	−0.5914	−2.1623
0.73	−2.9941	−1.6855	−2.2369	−2.5881	−2.5675	−0.7705	−0.5392	−0.9939	−2.1790	−1.6820	−2.0148	−0.8582	−0.1085	−1.8858
0.74	−3.2758	−1.4112	−1.6431	−1.9636	−2.2641	−0.0887	−0.4869	−0.6951	−1.7796	−0.9806	−1.9517	−0.7084	0.0198	−1.5117
0.75	−3.1215	−1.1479	−1.2102	−1.2181	−1.8310	0.0783	−0.4941	−0.6065	−1.2953	−0.5946	−1.8023	−0.6073	−0.2592	−1.1713
0.76	−2.5548	−0.9666	−0.9239	−0.7278	−1.5016	−0.2761	−0.6536	−0.5358	−0.9238	−0.4689	−1.4438	−0.6778	−0.4006	−1.0230
0.77	−1.9314	−0.8563	−0.8304	−0.6732	−1.2786	−0.5947	−0.6671	−0.5753	−0.6993	−0.4520	−1.1887	−0.8043	−0.4782	−0.9480
0.78	−1.4243	−0.6764	−0.8005	−0.8252	−1.0510	−0.8364	−0.5868	−0.5975	−0.5953	−0.5342	−1.1540	−0.9065	−0.6057	−0.9144
0.79	−1.1352	−0.5937	−0.8017	−0.9330	−1.0001	−0.8626	−0.6333	−0.5548	−0.6674	−0.5983	−1.1773	−1.0264	−0.6656	−0.9656
0.80	−1.0285	−0.5718	−0.7797	−0.8797	−1.0301	−0.6093	−0.6835	−0.5079	−0.7255	−0.6777	−1.2433	−1.1295	−0.7829	−1.0314
0.81	−0.8130	−0.5436	−0.7068	−0.8252	−1.0441	−0.6267	−0.7374	−0.4282	−0.7979	−0.7596	−1.1675	−1.1904	−0.9407	−1.0473
0.82	−0.4919	−0.6780	−0.6944	−0.9182	−1.0963	−0.8920	−0.8405	−0.4945	−0.8758	−0.7820	−0.9878	−1.1886	−0.9701	−1.0078
0.83	−0.3252	−0.7663	−0.7220	−1.1771	−1.1641	−0.9536	−0.9484	−0.6670	−0.8111	−0.8004	−0.9080	−1.1298	−0.9462	−0.9574
0.84	−0.4131	−0.7683	−0.7922	−1.3981	−1.2436	−0.9637	−1.0101	−0.7517	−0.7688	−0.7697	−0.8364	−1.0178	−0.8720	−0.8723
0.85	−0.7292	−0.8427	−0.9449	−1.4282	−1.3213	−1.0130	−0.9785	−0.8331	−0.7811	−0.7237	−0.8296	−0.8956	−0.7233	−0.7664
0.86	−1.0335	−0.8806	−1.0786	−1.3419	−1.3058	−1.0028	−0.9255	−0.8723	−0.7282	−0.7240	−0.7803	−0.8122	−0.5627	−0.7122
0.87	−1.1244	−0.9067	−1.1302	−1.1381	−1.2249	−0.9181	−0.8835	−0.8119	−0.7253	−0.6846	−0.6021	−0.7568	−0.4639	−0.6181
0.88	−1.0809	−0.9914	−1.1426	−0.9030	−1.1385	−0.8694	−0.8003	−0.7961	−0.6956	−0.6247	−0.5969	−0.6628	−0.4126	−0.5471
0.89	−1.0673	−1.0219	−1.0733	−0.8815	−1.0880	−0.8157	−0.7329	−0.7668	−0.5736	−0.5943	−0.6728	−0.4681	−0.4307	−0.6585
0.90	−1.1964	−1.0289	−1.0113	−0.8608	−1.0749	−0.7290	−0.7031	−0.6816	−0.5477	−0.5609	−0.6976	−0.3105	−0.4863	−0.7656
0.91	−1.4426	−1.0707	−1.0689	−0.7782	−1.0731	−0.6733	−0.6285	−0.6623	−0.5306	−0.5760	−0.8613	−0.3913	−0.5708	−0.8450
0.92	−0.7746	−0.7906	−0.7820	−0.8010	−0.8261	−0.7904	−0.7508	−0.5121	−0.4980	−0.4531	−0.5132	−0.4694	−0.4538	−0.4796
0.93	−0.0713	−0.5231	−0.5030	−0.8242	−0.5514	−0.8841	−0.9259	−0.3659	−0.5114	−0.3137	−0.0659	−0.5291	−0.3447	−0.0992
0.94	−1.1839	−2.7572	−2.3438	−3.5213	−3.5239	−2.1381	−1.4911	−1.9692	−1.8520	−1.5684	−1.9514	−2.9357	−0.8362	−2.9083
0.95	−0.4163	−0.5819	−0.6282	−0.7508	−0.4265	−0.7119	−0.9160	−0.3234	−0.4950	−0.3847	−0.1032	−0.8520	−0.5335	−0.3500

**Table A4:** Accelerations measured for tests from 43 to 48 and from 61 to 67.

Time in ms	7 442 HB						9 450 HB						
	Test 43	Test 44	Test 45	Test 46	Test 47	Test 48	Test 61	Test 62	Test 63	Test 64	Test 65	Test 66	Test 67
0.01	0.5310	1.5310	2.1721	1.3565	2.0705	1.0174	1.2351	1.6769	1.1465	1.0299	1.7893	1.7294	1.5844
0.02	1.7356	2.5026	3.3487	2.4904	3.0820	2.1811	2.3817	2.7515	2.1879	2.1175	2.7420	2.5872	2.4110
0.03	2.9221	3.6822	4.5714	3.6106	4.2566	3.2649	3.5420	3.8992	3.3134	3.1755	3.8927	3.6518	3.4974
0.04	4.1775	5.0672	5.8772	4.8586	5.6508	4.3506	4.6687	5.1921	4.5339	4.2971	5.2576	4.9039	4.7909
0.05	5.5557	6.2752	7.0595	6.1856	6.8724	5.6498	5.8069	6.3876	5.8430	5.6288	6.4882	5.9918	5.8755
0.06	6.8266	7.5220	8.1553	7.2884	8.0108	6.8175	6.9782	7.5100	7.1099	6.8062	7.6827	7.1242	7.0053
0.07	8.0409	8.7609	9.2392	8.4639	9.2085	7.9336	8.0732	8.7430	8.3056	7.9386	8.8760	8.2404	8.1743
0.08	9.1583	9.6139	10.0057	9.4733	9.9654	9.0802	9.0172	9.6041	9.3753	9.1082	9.6931	9.0320	8.9948
0.09	9.9602	10.3334	10.5934	10.0824	10.5869	9.8383	9.7856	10.2674	10.1888	9.8824	10.4420	9.7962	9.7746
0.10	10.5699	10.7938	10.9588	10.7267	11.0627	10.4817	10.3969	10.9195	10.8448	10.5534	11.0664	10.3034	10.3079
0.11	10.9085	11.0357	11.1224	11.0450	11.2198	10.8961	10.7780	11.1408	11.2622	11.0375	11.3201	10.5735	10.5466
0.12	11.1002	11.2840	11.3049	11.1726	11.4472	11.0634	11.0516	11.3237	11.4927	11.2242	11.5573	10.8244	10.8336
0.13	11.2510	11.3083	11.1987	11.4114	11.4438	11.3124	11.2508	11.4678	11.7170	11.4730	11.6300	10.8229	10.9428
0.14	11.1077	11.0301	10.7199	11.1844	10.9749	11.2203	11.0455	11.1179	11.6345	11.4486	11.3312	10.7048	10.8257
0.15	10.6523	10.3773	9.8069	10.5550	10.1731	10.7681	10.5730	10.4918	11.1612	11.0280	10.7318	10.2867	10.4677
0.16	9.6154	9.1790	8.2920	9.6267	8.8225	9.9967	9.7397	9.3935	10.2878	10.2929	9.5410	9.2538	9.5539
0.17	8.0625	7.6180	6.5559	8.2315	7.0863	8.6302	8.3727	7.7643	8.8118	8.9999	7.9072	7.9296	8.2933
0.18	6.3067	5.7791	4.7668	6.5654	5.2363	7.0501	6.9021	6.0132	7.0587	7.4167	6.1287	6.3031	6.8350
0.19	4.1982	3.6570	2.7757	4.4831	3.1475	5.3392	5.1896	3.9872	5.1296	5.6297	4.0798	4.4012	5.0256
0.22	2.0583	1.5591	0.7546	2.1798	1.0738	3.3165	3.1818	1.8810	2.9723	3.5540	1.9923	2.5553	3.1416
0.21	0.0348	−0.3919	−1.4602	0.1037	−0.9641	1.3775	1.3108	−0.0758	0.8804	1.5403	−0.0970	0.6622	1.2476
0.22	−2.2006	−2.3948	−3.7914	−1.9126	−3.1061	−0.6000	−0.6501	−2.2354	−1.2695	−0.5391	−2.3520	−1.3642	−0.8206
0.23	−4.2927	−4.4302	−5.9162	−3.7499	−5.0902	−2.7841	−2.6539	−4.2859	−3.4351	−2.7260	−4.3834	−3.2699	−2.7651
0.24	−6.2904	−6.5494	−7.8932	−5.4740	−7.0259	−4.8090	−4.4220	−6.1980	−5.4099	−4.6952	−6.3287	−5.2003	−4.6200
0.25	−8.4114	−8.6391	−9.6763	−7.4496	−8.9397	−6.8103	−6.3106	−8.2216	−7.4309	−6.6938	−8.3357	−7.1458	−6.5199
0.26	−10.1316	−10.4217	−11.2644	−9.1967	−10.5275	−8.7646	−8.1506	−9.8717	−9.3331	−8.6069	−10.0313	−8.8676	−8.2442
0.27	−11.6171	−11.9636	−12.7307	−10.6027	−11.9463	−10.3244	−9.7486	−11.2796	−10.9464	−10.1608	−11.5633	−10.4376	−9.8807
0.28	−12.9948	−13.1844	−13.8692	−11.9241	−13.1805	−11.6598	−11.2967	−12.6313	−12.4383	−11.6788	−12.8932	−11.7646	−11.3713
0.29	−13.9468	−14.1302	−14.6647	−12.7729	−13.9928	−12.7620	−12.5970	−13.5038	−13.5677	−12.9469	−13.7735	−12.8024	−12.5114
0.30	−14.6997	−14.9807	−15.2540	−14.0213	−14.6327	−13.5430	−13.5743	−14.2144	−14.3103	−13.8291	−14.4640	−13.6924	−13.4630
0.31	−15.1959	−15.4429	−15.5377	−15.4075	−15.0244	−14.1450	−14.3323	−14.7813	−14.8898	−14.5907	−14.9432	−14.2683	−14.1674
0.32	−15.1209	−15.4884	−15.5758	−15.2435	−14.8996	−14.4643	−14.6719	−14.8740	−15.1303	−14.9576	−15.0417	−14.4745	−14.5462
0.33	−14.4913	−15.0201	−14.8647	−13.9461	−14.0992	−14.4846	−14.5925	−14.5685	−15.0037	−14.9762	−14.6365	−14.3635	−14.6722
0.34	−12.9527	−13.5455	−12.9124	−11.6866	−12.3606	−13.8939	−13.8278	−13.4169	−14.2314	−14.5383	−13.3186	−13.5863	−14.1329
0.35	−10.4811	−11.2289	−10.0287	−8.3700	−9.7726	−12.2895	−12.1160	−11.3444	−12.5112	−13.1043	−11.1127	−12.0876	−12.7253
0.36	−7.4555	−8.2702	−6.4609	−5.5461	−6.5771	−9.9109	−9.8190	−8.6872	−10.0348	−10.9182	−8.1590	−10.0195	−10.6993
0.37	−4.1503	−4.8043	−2.8520	−3.0428	−3.1872	−7.0859	−7.0968	−5.4487	−6.9656	−8.1535	−4.7274	−7.3355	−8.0336
0.38	−1.1993	−1.5825	0.0460	−0.0991	−0.2162	−4.3241	−4.3130	−2.1102	−3.7431	−5.1090	−1.5228	−4.2196	−5.0760
0.39	1.0818	1.0908	2.3646	2.3090	2.1399	−2.0541	−1.9396	0.9225	−0.8238	−2.5009	1.1425	−1.0012	−2.3303
0.40	2.6315	3.0372	4.0174	3.9575	3.4711	−0.0736	0.1834	3.4007	1.7286	−0.1600	3.0658	1.9127	0.1819
0.41	3.2809	3.9331	4.4555	4.7520	4.2964	1.5749	1.8789	4.6664	3.5910	1.9210	3.8732	3.9384	2.1648
0.42	3.3618	3.9910	5.4872	4.7060	5.4704	2.7717	2.8973	5.0856	4.4557	3.4633	4.2547	4.8033	3.4630
0.43	3.1222	4.6972	5.9914	4.2058	5.2092	3.5734	3.4511	5.1718	5.2488	4.4542	4.8496	5.3312	4.1659
0.44	2.6413	5.0333	4.0372	3.3556	3.6195	4.9262	3.6465	4.4035	5.5220	4.9406	4.5361	5.1115	4.8530
0.45	2.2510	3.5760	2.2204	2.6300	2.5554	5.2410	3.9831	3.2825	4.4405	4.9390	3.3661	3.7749	4.9014
0.46	1.8296	2.0983	1.0659	2.1478	1.4078	3.5674	3.7780	2.3857	3.1586	3.8773	2.3561	2.5506	3.6273
0.47	1.3410	1.0648	−0.1054	1.6718	0.8879	2.0880	2.5717	1.5161	2.0702	2.2845	1.4283	1.5459	2.2876
0.48	0.9275	0.1555	0.5736	1.4810	1.6990	0.9067	1.6124	1.2118	1.0817	1.3647	1.0181	0.8419	1.3807
0.49	0.3920	0.7401	1.5505	1.3387	1.6663	0.0044	0.9790	1.4140	1.0568	0.8304	1.4449	1.2682	0.6831
0.50	−0.1619	1.5825	0.9543	0.9559	1.1702	0.8554	0.6107	1.4132	1.3798	0.8321	1.5780	1.7834	1.0383
0.51	−0.5984	1.1094	0.9648	0.5363	1.5010	1.5379	1.1898	1.4071	1.1932	1.5733	1.2979	1.6886	1.6158
0.52	−1.1009	1.0154	1.3570	−0.0350	1.6146	1.0373	1.5732	1.4702	1.2618	1.7357	1.3433	1.8536	1.4067
0.53	−1.5084	1.3160	1.3423	−0.5908	1.5286	1.5200	1.3704	1.4866	1.5007	1.6181	1.4761	1.9551	1.6062
0.54	−1.7937	1.3049	1.3512	−0.8554	1.5983	2.1441	1.6664	1.5524	1.5840	1.9860	1.4772	1.8009	1.9683
0.55	−2.0772	1.2418	1.4793	−1.1303	1.5357	2.1678	1.9385	1.4630	1.5342	2.0889	1.3997	1.6175	1.9202
0.56	−2.2322	1.3187	1.5753	−1.0672	1.4698	2.1874	1.9154	1.4699	1.4646	1.9019	1.3841	1.4908	1.8610
0.57	−2.3295	1.3201	1.5433	−0.2154	1.3653	2.1394	1.8790	1.5378	1.4743	1.8017	1.3524	1.4704	1.7301
0.58	−2.1174	1.2273	1.3784	−0.1578	1.1299	1.7775	1.6591	1.4106	1.3597	1.6237	1.2094	1.4486	1.4182
0.59	−1.5670	1.1304	1.1854	−0.9463	0.9875	1.4467	1.3554	1.4187	1.1614	1.4377	1.0885	1.4074	1.3205
0.60	−1.3067	0.9595	0.9536	−1.1736	0.8380	1.0283	1.0792	1.4221	1.0231	1.3223	0.9518	1.3843	1.2568
0.61	−1.2078	0.7357	0.6898	−1.2728	0.6605	0.5272	0.7991	1.2152	0.9186	1.1314	0.7236	1.3447	1.1372
0.62	−1.1191	0.5397	0.4638	−1.1101	0.5890	0.3329	0.6793	1.0741	0.9250	1.0150	0.5857	1.3767	1.1619
0.63	−1.2923	0.3195	0.1700	−0.2469	0.4522	0.3559	0.5324	0.8318	0.9591	1.0214	0.4836	1.3335	1.0938
0.64	−1.2239	0.1010	−0.0996	−0.0373	0.3180	0.5078	0.2941	0.5030	0.8162	1.0107	0.3535	1.1311	0.9729
0.65	−0.9537	−0.0332	−0.1910	−0.4493	0.3056	0.7660	0.1904	0.3501	0.6484	0.9363	0.3191	0.8805	0.9151
0.66	−0.9679	−0.1497	−0.2319	−0.2914	0.2319	0.8493	0.0520	0.1628	0.5410	0.7154	0.2678	0.5553	0.6795
0.67	−0.9464	−0.2161	−0.2455	−0.1145	0.2250	0.8442	−0.0367	−0.0164	0.4559	0.4903	0.2056	0.2828	0.4582
0.68	−0.9299	−0.2360	−0.2018	−0.3420	0.2625	0.8222	0.0704	−0.0591	0.4445	0.5054	0.2633	0.1832	0.4127
0.69	−1.1066	−0.2547	−0.2204	−0.6407	0.2168	0.6357	0.0866	−0.1655	0.3513	0.5894	0.2977	0.0857	0.3995
0.70	−1.2470	−0.2385	−0.2151	−0.7358	0.2688	0.4816	0.1108	−0.1667	0.1520	0.5905	0.3166	0.0267	0.4909
0.71	−1.3604	−0.1973	−0.1064	−0.8421	0.3276	0.3577	0.2217	−0.0261	0.0410	0.4669	0.3752	0.0532	0.4628
0.72	−1.4420	−0.1479	0.0049	−0.9003	0.2999	0.2074	0.1784	0.0445	0.0219	0.2396	0.3432	0.0430	0.2907
0.73	−1.3854	−0.0820	0.1003	−0.8521	0.3033	0.2055	0.1484	0.1906	0.0216	0.1819	0.3329	0.1032	0.2777
0.74	−1.3334	−0.0294	0.1072	−0.8612	0.2443	0.1808	0.1918	0.3027	0.0480	0.3153	0.3241	0.2594	0.3162
0.75	−1.2965	0.0034	−0.0113	−0.7844	0.1888	0.0944	0.1638	0.3006	0.0000	0.3865	0.2551	0.3983	0.3852
0.76	−1.2220	−0.0125	−0.1315	−0.5636	0.2353	0.1224	0.2008	0.3775	−0.0444	0.3882	0.2332	0.5289	0.4934
0.77	−1.1536	−0.0724	−0.1690	−0.3459	0.2056	0.1080	0.1494	0.3979	0.0154	0.3064	0.1992	0.5709	0.3828
0.78	−1.1054	−0.0994	−0.1753	−0.1244	0.1698	0.1591	0.0004	0.3466	0.0656	0.1637	0.1477	0.5037	0.2545
0.79	−1.0348	−0.1275	−0.1945	0.0372	0.1638	0.3234	−0.0179	0.3594	0.1458	0.1495	0.1381	0.4437	0.2451
0.80	−0.9309	−0.1944	−0.2523	0.0555	0.0791	0.3891	−0.0413	0.2802	0.2204	0.2205	0.0968	0.3760	0.2237
0.81	−0.8782	−0.2559	−0.3674	0.1163	0.0722	0.4548	−0.0674	0.2077	0.2112	0.2548	0.0740	0.2840	0.2758
0.82	−0.8799	−0.3127	−0.4610	0.4002	0.1045	0.4923	−0.0123	0.1864	0.2429	0.2234	0.0960	0.2210	0.2685
0.83	−0.8652	−0.3641	−0.4403	0.6187	0.0414	0.3792	−0.0126	0.0569	0.2957	0.1046	0.1020	0.1198	0.1114
0.84	−0.8422	−0.3873	−0.3571	0.4492	0.0380	0.3017	0.0348	−0.0082	0.2897	0.0182	0.1368	−0.0272	0.0232
0.85	−0.8763	−0.3833	−0.2902	0.1346	0.0564	0.2063	0.1649	−0.0158	0.3119	0.0232	0.1762	−0.1002	−0.0159
0.86	−0.8791	−0.3871	−0.2487	−0.1341	0.0315	0.0833	0.2183	−0.0681	0.2728	0.0789	0.2013	−0.1310	−0.0198
0.87	−0.8734	−0.3662	−0.2147	−0.2944	0.1040	0.0703	0.2799	−0.0392	0.1845	0.1270	0.2709	−0.1525	0.0871
0.88	−0.9283	−0.2920	−0.2097	−0.1851	0.1461	−0.0070	0.3044	−0.0058	0.1656	0.0794	0.3160	−0.1169	0.1166
0.89	−0.9281	−0.2369	−0.2222	−0.0814	0.1370	−0.1346	0.2705	−0.0065	0.1234	0.0024	0.3142	−0.0747	0.0861
0.90	−0.9274	−0.2369	−0.2154	−0.2295	0.1722	−0.1827	0.2658	0.0848	0.0775	−0.0052	0.3373	−0.0433	0.1054
0.91	−1.0244	−0.2696	−0.3018	−0.3589	0.1075	−0.2981	0.2243	0.1163	0.0382	−0.0643	0.3213	0.0017	0.0573
0.92	−0.7310	−0.0159	−0.0094	0.0099	0.2357	0.2113	0.1953	0.2334	0.2614	0.2367	0.2566	0.2608	0.2466
0.93	−0.4258	0.2120	0.3018	0.3850	0.4138	0.7864	0.1931	0.4007	0.4622	0.5866	0.2116	0.5126	0.4695
0.94	−11.7999	1.6557	1.2981	−9.6857	2.2359	2.6358	0.2286	2.3493	2.2694	3.2621	2.4877	3.6893	3.2959
0.95	−0.4979	0.1323	0.0871	0.3547	0.3024	0.7396	0.1439	0.4634	0.2935	0.5731	0.1135	0.6909	0.4391

**Table A5:** Accelerations measured for tests from 49 to 60.

Time in ms	8 446 HB											
	Test 49	Test 50	Test 51	Test 52	Test 53	Test 54	Test 55	Test 56	Test 57	Test 58	Test 59	Test 60
0.01	1.3368	1.7362	1.4114	2.1965	1.1621	1.9785	1.5047	1.2461	1.9580	2.1968	1.4002	1.8195
0.02	2.5311	2.7178	2.3282	3.1953	2.1926	2.9612	2.5334	2.2980	2.9030	3.2490	2.5555	2.7753
0.03	3.7230	3.9426	3.4086	4.3761	3.2903	4.1411	3.7258	3.4022	4.0579	4.4176	3.6635	3.9213
0.04	5.0490	5.3770	4.6977	5.7456	4.4951	5.5248	5.0831	4.5969	5.4006	5.6583	4.8731	5.2761
0.05	6.5246	6.6372	5.9614	6.8741	5.7438	6.6436	6.2919	5.8612	6.5652	6.8206	6.2016	6.4083
0.06	7.7362	7.8462	7.1852	8.0246	6.9424	7.7668	7.4399	7.0501	7.7195	7.9714	7.3185	7.5279
0.07	8.9419	9.0832	8.4201	9.1813	8.1568	8.9140	8.6059	8.1897	8.8162	9.0170	8.4774	8.7309
0.08	9.9822	9.9305	9.4248	9.8596	9.1940	9.6242	9.4795	9.2161	9.6244	9.7505	9.4557	9.5066
0.09	10.6025	10.6508	10.2483	10.4866	9.9750	10.3312	10.2030	9.9472	10.3692	10.3464	10.0592	10.1810
0.10	11.2569	11.1741	10.8351	10.8864	10.6118	10.7982	10.7700	10.5230	10.7320	10.7039	10.6901	10.6699
0.11	11.5855	11.3744	11.1594	10.9594	10.8958	10.9530	11.0240	10.8535	10.9169	10.9069	10.9048	10.8004
0.12	11.7154	11.6038	11.4092	11.2033	11.0967	11.1881	11.1733	10.9906	11.1451	11.1075	11.0172	10.9874
0.13	11.9057	11.6738	11.5060	11.1557	11.2994	11.1546	11.2170	11.1557	11.0605	10.9915	11.2386	10.9933
0.14	11.6038	11.4129	11.4325	10.6640	11.1818	10.7721	10.9549	10.9936	10.8232	10.6012	10.9198	10.6398
0.15	10.8834	10.7277	10.9821	9.9097	10.8014	10.0730	10.3699	10.4562	10.1476	9.7911	10.3654	10.0347
0.16	9.7415	9.4165	9.8975	8.4837	9.8933	8.7889	9.3037	9.5468	8.7938	8.4207	9.3889	8.8890
0.17	8.0190	7.7091	8.4692	6.7755	8.4610	7.2033	7.7588	8.1091	7.2668	6.8348	7.7254	7.3354
0.18	6.1813	5.8239	6.7188	4.9707	6.8646	5.4476	6.0341	6.4433	5.4881	4.9623	5.9900	5.5522
0.19	4.1664	3.7237	4.6979	2.7793	4.9477	3.3917	4.0667	4.5284	3.4270	2.8829	3.9941	3.4251
0.22	1.9578	1.6212	2.6976	0.7291	2.8982	1.3729	1.9649	2.4137	1.4714	0.9122	1.8344	1.3561
0.21	−0.1471	−0.4901	0.5960	−1.3079	0.9624	−0.6765	−0.0737	0.4149	−0.6101	−1.1539	−0.0784	−0.5742
0.22	−2.3333	−2.7169	−1.6519	−3.5469	−1.1483	−2.8489	−2.2823	−1.6875	−2.7580	−3.2080	−2.1837	−2.6297
0.23	−4.5081	−4.7939	−3.7810	−5.4371	−3.1838	−4.7897	−4.3839	−3.7608	−4.6392	−5.0966	−4.2494	−4.5436
0.24	−6.5570	−6.7873	−5.8813	−7.3408	−5.0976	−6.6682	−6.2325	−5.6460	−6.5340	−7.0332	−6.0996	−6.5083
0.25	−8.6581	−8.7431	−7.9394	−9.2192	−7.1462	−8.4820	−8.0822	−7.6120	−8.3802	−8.8157	−8.0827	−8.5549
0.26	−10.5029	−10.4048	−9.6892	−10.6412	−8.9526	−9.9503	−9.7316	−9.3686	−9.9961	−10.3661	−9.8059	−10.1655
0.27	−11.9703	−11.8805	−11.2631	−12.0583	−10.5308	−11.2722	−11.1963	−10.8236	−11.5457	−11.8200	−11.2611	−11.5443
0.28	−13.2778	−13.1101	−12.6393	−13.2174	−11.9998	−12.3637	−12.5144	−12.2242	−12.7869	−12.9506	−12.6347	−12.7021
0.29	−14.2368	−13.9645	−13.6365	−13.8981	−13.0962	−13.1626	−13.4253	−13.2752	−13.6495	−13.7947	−13.5518	−13.4438
0.30	−14.8908	−14.6316	−14.4063	−14.5481	−13.9548	−13.9051	−14.0958	−14.0007	−14.3523	−14.4265	−14.2297	−14.1194
0.31	−15.3502	−15.0110	−14.9110	−14.8519	−14.5939	−14.3221	−14.5647	−14.5813	−14.6943	−14.6875	−14.7283	−14.5647
0.32	−15.3002	−15.0423	−15.0421	−14.6503	−14.8120	−14.3867	−14.6706	−14.7819	−14.7346	−14.6000	−14.7897	−14.5049
0.33	−14.5442	−14.5356	−14.7626	−13.8014	−14.6860	−13.9175	−14.2672	−14.5211	−14.2328	−13.8708	−14.4049	−13.8879
0.34	−12.9696	−13.0498	−13.6624	−11.8941	−13.8030	−12.4637	−12.9277	−13.4932	−12.6894	−12.1522	−13.0684	−12.3315
0.35	−10.5125	−10.8204	−11.7157	−9.2286	−11.9680	−10.2790	−10.6849	−11.4227	−10.3544	−9.5601	−10.6284	−9.9742
0.36	−7.3775	−8.0354	−9.1531	−6.0056	−9.4490	−7.4946	−7.8054	−8.4693	−7.4116	−6.1450	−7.4570	−7.1329
0.37	−4.0122	−4.8639	−6.1511	−2.6207	−6.2227	−4.3475	−4.5607	−4.8935	−4.1729	−2.4848	−3.8126	−4.0093
0.38	−0.9162	−1.8166	−3.1166	0.2017	−2.9122	−1.5155	−1.5980	−1.4080	−1.2597	0.6208	−0.4355	−1.1067
0.39	1.6370	0.9187	−0.2211	2.4815	−0.1159	0.8476	0.8630	1.4551	1.2910	3.0050	2.1563	1.3953
0.40	3.4081	3.0562	2.2305	3.7190	2.1322	2.5874	2.8113	3.5551	3.0722	4.1273	3.8931	3.2397
0.41	4.1826	4.1981	3.7578	4.3422	3.5056	3.6055	3.8680	4.4410	4.0321	4.7417	4.3672	4.1358
0.42	4.5483	4.5249	4.4236	5.4565	3.8111	4.4258	4.3567	4.7237	5.5037	5.7208	5.1260	5.5662
0.43	4.5872	4.9506	5.2383	5.1269	4.1623	5.0816	5.2726	5.1200	5.6040	5.0361	5.5562	5.9627
0.44	3.6547	5.3066	5.4039	3.3932	4.6931	4.7785	5.1153	4.4380	3.8116	3.1539	3.9342	4.1191
0.45	2.5453	4.4579	4.1415	2.2800	4.0980	3.6027	3.3788	3.0239	2.6493	1.9994	2.3544	2.6528
0.46	1.8468	2.9619	2.7900	1.1325	2.8614	2.3936	1.9624	2.0328	1.5932	0.8338	1.3625	1.4906
0.47	1.2647	1.7357	1.6678	0.6827	1.9091	1.5324	0.9383	1.1089	0.8454	0.5821	0.3574	0.4992
0.48	1.3149	0.7260	0.8012	1.6514	0.9867	1.3017	0.4483	0.8810	1.6974	1.6626	0.9564	1.4167
0.49	1.6963	0.6582	1.1445	1.7349	0.8106	1.6533	1.3073	1.5298	1.8932	1.7218	1.7995	2.1035
0.50	1.5928	1.2450	1.5931	1.3031	1.4457	1.7222	1.8630	1.6413	1.2183	1.3448	1.2965	1.5068
0.51	1.5735	1.1796	1.2166	1.6397	1.4619	1.2962	1.4484	1.4203	1.5343	1.6700	1.3395	1.7626
0.52	1.7305	1.0197	1.2586	1.7380	1.1867	1.0556	1.5022	1.5472	1.7418	1.7109	1.6551	2.0010
0.53	1.6317	1.2986	1.5889	1.6965	1.3407	1.0703	1.7048	1.5979	1.6089	1.6364	1.6057	1.7863
0.54	1.5025	1.4514	1.6807	1.6902	1.4537	1.0857	1.6198	1.6263	1.4731	1.6302	1.5990	1.6010
0.55	1.3557	1.4082	1.6110	1.5468	1.3601	1.0784	1.3952	1.6246	1.3338	1.5710	1.5237	1.3685
0.56	1.2403	1.4106	1.5405	1.4615	1.3542	1.0912	1.2715	1.5763	1.3666	1.4883	1.4823	1.1722
0.57	1.1911	1.3576	1.3771	1.2791	1.3450	0.9935	1.1518	1.5000	1.3277	1.2449	1.4619	1.0420
0.58	0.9917	1.1826	1.1586	1.0544	1.2167	0.9211	0.9870	1.3156	1.1225	0.9880	1.2176	0.8604
0.59	0.7661	1.0006	1.0157	0.9765	1.0929	0.9968	0.9017	1.1631	0.9791	0.8446	0.9757	0.7442
0.60	0.6674	0.8115	0.8490	0.7698	0.9430	0.9692	0.7658	1.0173	0.8457	0.6606	0.7786	0.6211
0.61	0.5341	0.6354	0.6592	0.5877	0.7373	0.8358	0.5669	0.7653	0.8096	0.4987	0.5361	0.5323
0.62	0.4400	0.5735	0.5620	0.5320	0.5960	0.6990	0.4248	0.5596	0.8448	0.3785	0.4377	0.5888
0.63	0.3701	0.5778	0.4575	0.3394	0.4217	0.5593	0.3326	0.3719	0.7139	0.1836	0.3266	0.5820
0.64	0.2051	0.6245	0.4379	0.2497	0.2718	0.5458	0.3028	0.1753	0.5318	0.1190	0.1356	0.5214
0.65	0.1483	0.6076	0.5047	0.2552	0.2210	0.5622	0.3291	0.1246	0.4124	0.1414	0.0786	0.4970
0.66	0.1661	0.4807	0.4860	0.1483	0.1755	0.4355	0.3230	0.0623	0.3319	0.1589	0.0204	0.3722
0.67	0.1300	0.3899	0.4420	0.1561	0.1953	0.3195	0.2591	−0.0225	0.3564	0.2358	0.0034	0.2874
0.68	0.1619	0.3329	0.3841	0.1851	0.2582	0.2383	0.1631	0.0184	0.3508	0.2676	0.0544	0.2849
0.69	0.1890	0.3174	0.2881	0.1380	0.2714	0.1678	0.0803	0.0665	0.2965	0.2560	0.0284	0.1870
0.70	0.1869	0.3361	0.2652	0.2042	0.2877	0.2410	0.0527	0.1064	0.3168	0.3093	0.0414	0.1375
0.71	0.2831	0.2290	0.2630	0.2263	0.3419	0.3081	0.0695	0.1683	0.3289	0.3226	0.1263	0.1373
0.72	0.3154	0.0776	0.1979	0.2271	0.3771	0.2715	0.0517	0.1471	0.4065	0.3268	0.1191	0.1064
0.73	0.3285	0.0382	0.1366	0.2730	0.3980	0.2184	0.0124	0.1517	0.5146	0.3631	0.1652	0.1513
0.74	0.3393	0.0493	0.0888	0.1739	0.3859	0.1344	0.0296	0.2070	0.4463	0.2850	0.1847	0.1373
0.75	0.2393	0.1356	0.1127	0.1037	0.3309	0.0950	0.0606	0.1863	0.3617	0.1801	0.1186	0.0390
0.76	0.1850	0.1913	0.1880	0.1136	0.2729	0.0937	0.1220	0.1973	0.3387	0.1028	0.1214	0.0081
0.77	0.1644	0.1096	0.2016	0.0273	0.2298	0.0184	0.1566	0.1735	0.2441	−0.0007	0.1075	−0.0405
0.78	0.0922	0.0382	0.1600	0.0055	0.1896	−0.0502	0.0983	0.0380	0.2159	−0.0355	0.0744	−0.0338
0.79	0.0687	0.0472	0.0972	0.0168	0.1594	−0.0652	0.0929	−0.0239	0.1850	−0.0201	0.1092	0.0625
0.80	0.0093	0.0972	0.0445	−0.0594	0.1211	−0.0574	0.1661	−0.0380	0.0531	−0.0504	0.0665	0.0448
0.81	−0.0960	0.1778	0.0862	−0.0437	0.1143	0.0245	0.2464	−0.0780	0.0244	−0.0365	0.0090	0.0221
0.82	−0.0885	0.1977	0.1439	−0.0434	0.1334	0.0712	0.2985	−0.0370	0.0339	0.0045	0.0150	0.0412
0.83	−0.0851	0.1592	0.1215	−0.1109	0.1354	0.0813	0.2046	−0.0229	0.0157	0.0266	−0.0283	−0.0053
0.84	−0.0753	0.1273	0.0981	−0.0992	0.1566	0.1687	−0.0068	−0.0479	0.0655	0.0906	0.0188	0.0184
0.85	−0.0091	0.1025	0.0683	−0.0927	0.1921	0.2695	−0.1347	0.0007	0.0890	0.1126	0.0625	0.0591
0.86	0.0454	0.1184	0.0587	−0.0711	0.2365	0.3442	−0.1382	0.0201	0.1186	0.1244	0.0121	0.0349
0.87	0.1239	0.1435	0.0999	0.0203	0.2721	0.3697	−0.0696	0.0161	0.2078	0.1760	0.0218	0.0793
0.88	0.1990	0.1703	0.1136	0.0552	0.2531	0.3072	0.0308	0.0527	0.2522	0.1682	0.0387	0.1046
0.89	0.2380	0.2266	0.1123	0.1001	0.2338	0.2269	0.0915	0.0495	0.2989	0.1730	0.0106	0.0846
0.90	0.2548	0.2606	0.0840	0.1602	0.2200	0.1874	0.1101	0.0568	0.3113	0.1740	0.0742	0.1208
0.91	0.2088	0.2221	−0.0060	0.1222	0.1965	0.1359	0.1190	0.0150	0.2340	0.0878	0.0862	0.0530
0.92	0.2115	0.2347	0.2157	0.2006	0.2237	0.2238	0.2023	0.2447	0.2549	0.2159	0.2016	0.2163
0.93	0.2478	0.2350	0.4584	0.3145	0.2678	0.3179	0.2907	0.4755	0.2950	0.3866	0.3739	0.4242
0.94	0.5909	1.4713	1.3054	0.6648	2.0111	1.7833	0.4078	1.2812	3.0372	1.9155	0.7339	1.2923
0.95	0.1430	0.1645	0.3562	0.2893	0.3127	0.3147	0.3331	0.2346	0.1471	0.3958	0.3659	0.2546
